# PEAR1 Promotes Glucose Metabolism Reprogramming in Sepsis‐Associated Acute Lung Injury via AARS1‐Mediated HIF‐1α Lactylation

**DOI:** 10.1002/advs.76592

**Published:** 2026-07-14

**Authors:** Shuai Li, Zongqing Lu, Pu Fang, Qingqing Pang, Xianghui Wu, Bo Wang, Min Liang, Jingjing Pan, Haobo Kong, Haijian Cai, Juanjuan Li, Jiaying Kang, Dongsheng Wang, Dunling Xia, Mengmeng Xu, Qinghai You

**Affiliations:** ^1^ Department of Respiratory and Critical Care Medicine The First Affiliated Hospital of Anhui Medical University Hefei China; ^2^ Department of Intensive Care Unit The First Affiliated Hospital of Anhui Medical University Hefei China; ^3^ Department of Respiratory Intensive Care Unit Anhui Chest Hospital Hefei China; ^4^ Center For Scientific Research Anhui Medical University Hefei China; ^5^ Department of Pulmonary and Critical Care Medicine, Division of Life Sciences and Medicine, The First Affiliated Hospital of USTC University of Science and Technology of China Hefei China; ^6^ Department of Emergency Medicine The First Affiliated Hospital of Anhui Medical University Hefei China; ^7^ Laboratory of Structural Immunology, National Key Laboratory of Immune Response and Immunotherapy, Division of Life Sciences and Medicine University of Science and Technology of China Hefei China; ^8^ Anhui Provincial Key Laboratory of Geriatric Immunotherapy and Nutrition Therapy, Division of Life Sciences and Medicine, The First Affiliated Hospital of USTC University of Science and Technology of China Hefei China

**Keywords:** glycolysis, lactylation, PEAR1, sepsis‐associated acute lung injury

## Abstract

Sepsis‐associated acute lung injury (S‐ALI), in which pulmonary microvascular endothelial cells act as key drivers of disease progression by increasing vascular permeability and ultimately exacerbating lung injury, is associated with a high mortality rate. Here, we report that PEAR1 expression and vascular permeability are increased in the lung tissues of septic mice. *Pear1* knockdown markedly reduces pulmonary vascular permeability and consequently attenuates lung injury in septic mice. Mechanistically, PEAR1 promotes the AARS1‐mediated lactylation of HIF‐1α, primarily at lysine 172 (K172). This lactylation event, in turn, increases the affinity of HIF‐1α for importin α, thereby facilitating HIF‐1α nuclear translocation. Importantly, HIF‐1α K172 lactylation promotes glycolysis, and glycolysis‐derived lactate further drives H3K18 lactylation. In addition, this lactate‐dependent histone modification is enriched at the *Pear1* promoter, resulting in further increases in glycolysis and pulmonary vascular permeability. In vivo, both *Pear1* knockout and the targeted delivery of *Pear1* siRNA to inflammatory vascular endothelial cells using E‐selectin‐binding peptide‐modified liposomes ameliorate ALI, and improve survival in mice with polymicrobial sepsis. Our study identifies K172 as a previously unreported lactylation site on HIF‐1α and shows that PEAR1 promotes AARS1‐mediated HIF‐1α lactylation, enhances glycolysis, and increases H3K18la enrichment at the *Pear1* promoter, thereby forming a positive feedback loop.

## Graphical abstract

This study revealed that a PEAR1/HIF‐1α/ glycolysis/lactate/H3K18la positive feedback loop in PMVECs that drives the development of S‐ALI. Mechanistically, PEAR1 mediates the binding of HIF‐1α to AARS1, leading to the lactylation of HIF‐1α, the primary lactylation site of which is K172. K172 lactylation of HIF‐1α promotes its interaction with importin α, thereby promoting HIF‐1α nuclear localization. Significantly, enhanced nuclear localization of HIF‐1α stimulates glycolysis, and the lactate produced thereby increases H3K18 lactylation. This leads to H3K18la enrichment at the *Pear1* promoter, creating a positive feedback cycle that augments both glycolysis and pulmonary vascular permeability.

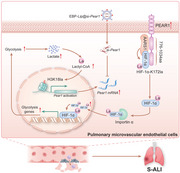

## 1 Introduction

Sepsis is a dysregulated host response to infection that leads to life‐threatening organ dysfunction [1, 2]. As a leading cause of hospital mortality and morbidity worldwide, sepsis is a critical global health challenge. Sepsis‐associated acute lung injury (S‐ALI) is a common and devastating complication of sepsis, with reported mortality rates ranging from 30% to 40% [3]. Pulmonary microvascular endothelial cells (PMVECs) not only serve as active mediators of S‐ALI but also represent primary sites of injury. PMVEC injury leads to increased vascular permeability, which causes inflammatory cell infiltration and further exacerbates damage [4, 5]. Consequently, therapeutic strategies targeting the preservation of microvascular endothelial function represent crucial intervention points for improving outcomes in S‐ALI patients.

Platelet endothelial aggregation receptor 1 (PEAR1) is a newly discovered membrane protein comprising 1034 amino acids, with repeated extracellular epidermal growth factor (EGF)‐like domains and multiple cytoplasmic tyrosine residues. PEAR1 was first reported in 2005 and is described as a platelet contact activation receptor that is expressed primarily on endothelial cells (ECs) and platelet membranes [6]. Although most studies to date have focused on the role of this receptor in platelets, PEAR1 is also expressed in ECs [6, 7]. Research has shown that knocking down *Pear1* promotes the proliferation of ECs through the Akt/p21/CDC2 signaling axis [8]. A previous study revealed that PEAR1 expression is significantly elevated in the lung tissues of S‐ALI mice. In addition, PEAR1 is involved in the negative regulation of human pulmonary microvascular endothelial cells (HPMECs) proliferation. *PEAR1* knockdown increases HPMECs proliferation, whereas *PEAR1* overexpression suppresses the proliferation of these cells [9]. More importantly, an increasing number of recent studies have reported that PEAR1 is involved in the regulation of lung injury induced by various stimuli [9, 10]. These findings suggest that PEAR1 may serve as a potential therapeutic target for S‐ALI. However, the mechanisms underlying PEAR1 upregulation and its downstream injury‐promoting effects during S‐ALI remain unclear.

Glycolysis is the primary pathway for ATP production in ECs. During sepsis, glucose metabolic reprogramming further enhances glycolysis, resulting in the conversion of nearly all glucose into lactate in ECs [11, 12]. Importantly, elevated serum lactate is considered not only a prognostic biomarker for sepsis but also an important signaling molecule that directly promotes the release of inflammatory factors, such as tumor necrosis factor‐α (TNF‐α) and interleukin‐6 (IL‐6), and induces sublethal endothelial injury, including loss of cell polarity and reduced expression of tight junction proteins, thereby contributing to endothelial dysfunction [13, 14]. In contrast, inhibiting glycolysis can effectively alleviate inflammation and improve sepsis prognosis, as demonstrated in experimental models of S‐ALI [15, 16]. Therefore, regulating metabolic reprogramming in PMVECs may be a promising strategy for treating S‐ALI.

Protein post‐translational modifications (PTMs) include phosphorylation, acetylation, methylation, and other types of modifications. These modifications are essential for controlling protein function and stability and constitute an integral component of the hierarchical architecture of epigenetic regulation [17, 18]. Notably, lactate, a metabolic byproduct of glycolysis, has been identified as a substrate for histone lactylation and can directly promote downstream gene transcription [19, 20]. Consistent with these findings, histone lactylation has emerged as an important epigenetic mechanism involved in disease pathogenesis [21, 22, 23]. For instance, in hepatic stellate cells, suppression of lactate production, achieved through either hexokinase 2 deletion or pharmacological intervention, markedly reduces histone lactylation, thereby attenuating cellular activation and ameliorating liver fibrosis [24]. Our previous investigation demonstrated that glycolysis‐derived lactate induces histone H3 lysine 18 lactylation (H3K18la) in the context of S‐ALI. This epigenetic modification induces the activation of the EGR1/HPSE signaling cascade, ultimately leading to degradation of the endothelial glycocalyx barrier [25]. However, the functional role of histone lactylation in the development of S‐ALI has not been fully elucidated.

Our results identify PEAR1 as a key endothelial regulator that is upregulated during S‐ALI and promotes pulmonary microvascular hyperpermeability. Mechanistically, PEAR1 acts through its intracellular domain to facilitate the interaction between AARS1 and HIF‐1α, leading to AARS1‐mediated lactylation of HIF‐1α at lysine 172 (K172), a previously unreported lactylation site. This modification enhances the interaction between HIF‐1α and importin α, thereby promoting HIF‐1α nuclear translocation and glycolytic reprogramming in PMVECs. The resulting increase in glycolysis‐derived lactate further induces H3K18 lactylation at the *Pear1* promoter, thereby enhancing *Pear1* transcription and establishing a PEAR1‐centered positive feedback loop that aggravates endothelial barrier dysfunction during S‐ALI. *Pear1* knockout or the selective delivery of *Pear1* small interfering RNA (siRNA) to inflammatory vascular ECs using E‐selectin‐binding peptide‐modified liposomes alleviates pulmonary vascular permeability, revealing a new mechanism and potential therapeutic target for S‐ALI.

## 2 Results

### 2.1 PEAR1 Is Upregulated in Lung Tissue and PMVECs After S‐ALI

**FIGURE 1:**
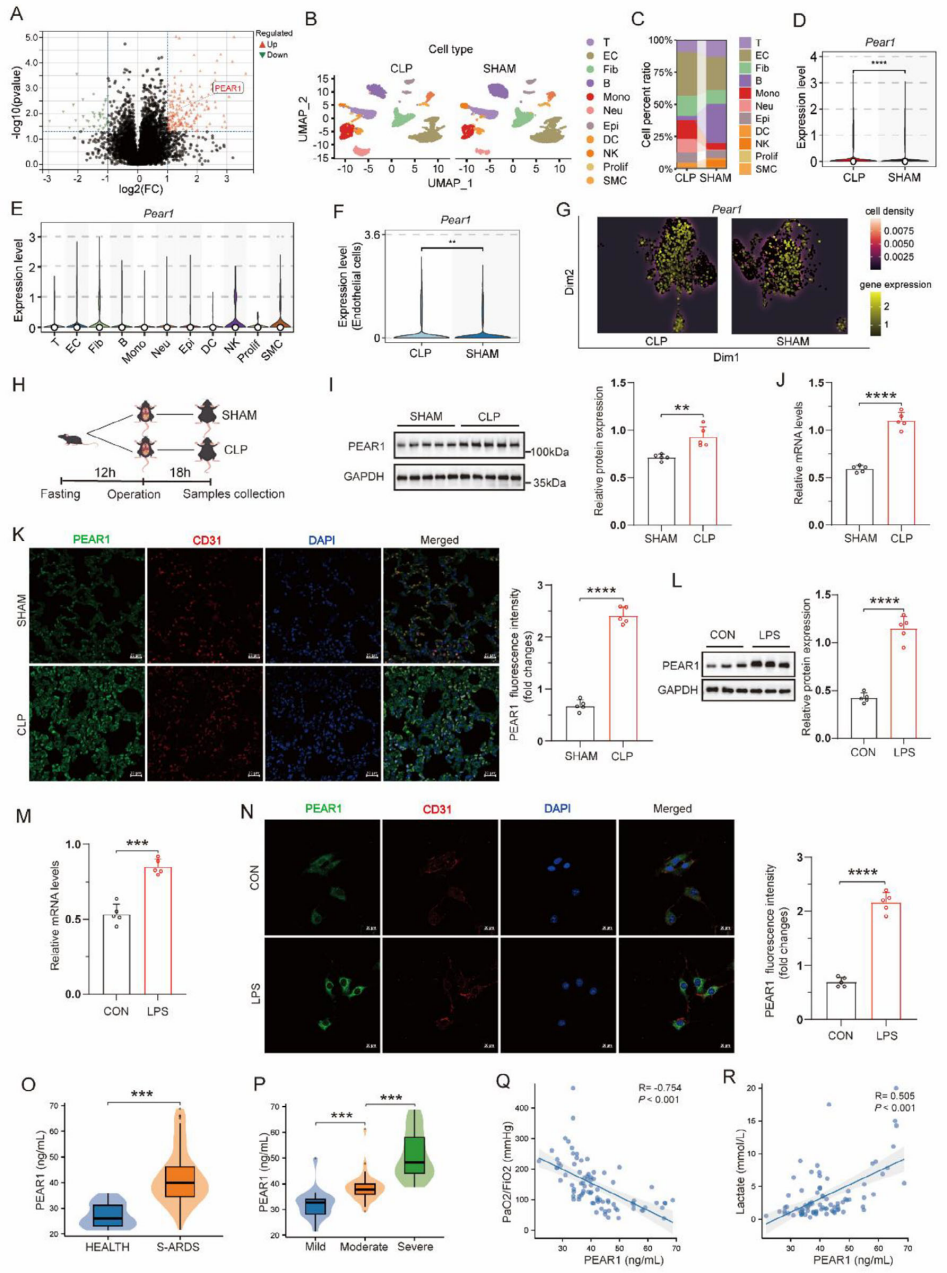
PEAR1 Is Upregulated in Lung Tissue and PMVECs After S‐ALI. (A) The lung tissue of mice in the SHAM and CLP group was subjected to proteomics and the results displayed in the form of a volcano plot. Screening criteria based on |log2(fold change (FC)| > 1 and *p* < 0.05 (n = 3 per group). (B) UMAP plot of 11 cell types identified from the lung tissue of SHAM and CLP group, including T cells, B cells, endothelial cells, fibroblasts, monocytes, neutrophils, epithelial cells, dendritic cells, NK cells, smooth muscle cells, and proliferating cells (n = 1 per group). (C) Proportion distribution of cell composition in lung tissue of two groups of mice. (D) The expression of *Pear1* in lung tissue of two groups of mice. (E) The expression of *Pear1* in different types of cells. (F) The expression of *Pear1* in lung endothelial cells of two groups of mice. (G) UMAP visualization of *Pear1* distribution in lung endothelial cells of two groups of mice. (H) Diagram of animal experimental procedures. (I) WB analysis of PEAR1 protein levels in lung tissue 18 h after SHAM or CLP surgery (n = 5 per group). (J) RT‐qPCR analysis of *Pear1* mRNA levels in lung tissue 18 h after Sham or CLP surgery (n = 5 per group). (K) Representative IF staining images and relative intensity quantification of PEAR1 (green) in lung tissues from SHAM and CLP groups (n = 5 per group). Vascular endothelium was labeled with CD31 (red), and nuclei were stained with DAPI (blue). Scale bar, 20 µm. (L) WB analysis of PEAR1 protein levels in MPMVECs with or without LPS stimulation (10 µg/mL for 12 h) (n = 5 per group). (M) RT‐qPCR analysis of *Pear1* mRNA levels in MPMVECs with or without LPS stimulation (10 µg/mL for 12 h) (n = 5 per group). (N) Representative IF staining images and relative intensity quantification of PEAR1 (green) in MPMVECs with or without LPS stimulation (10 µg/mL for 12 h) (n = 5 per group). MPMVECs surfaces were labeled with CD31 (red), and nuclei were stained with DAPI (blue). Scale bar, 20 µm. (O) Comparison of serum PEAR1 concentration between health controls and S‐ARDS patients. (P) Comparison of serum PEAR1 concentrations among patients with mild, moderate, and severe S‐ARDS. (Q, R) Correlation analysis of serum PEAR1 concentration with PaO_2_/FiO_2_ and blood lactate levels in S‐ARDS patients. All data were represented as the means ± SD, *p < 0.05, **p < 0.01, ***p < 0.001, and ****p < 0.0001; ns, not significant.

To identify potential regulators involved in S‐ALI, we performed proteomic analysis using a cecal ligation and puncture (CLP)‐induced murine lung injury model. Based on the predefined screening criteria of |log2(fold change [FC])| > 1 and *p* < 0.05, we identified 165 upregulated proteins and 38 downregulated proteins, among which PEAR1 protein expression was significantly increased (Figure 1A). Although PEAR1 was not the top‐ranked protein in terms of fold change or statistical significance, it was prioritized for further investigation because of its known biological relevance to vascular endothelial regulation, platelet‐endothelial interactions, inflammatory vascular responses, and lung injury [6, 8, 9, 10].

Subsequently, single‐cell RNA sequencing was performed to investigate the distribution of *Pear1* in lung tissues of septic mice. UMAP analysis identified 11 distinct cell types in mouse lungs, including T cells, B cells, endothelial cells, fibroblasts, monocytes, neutrophils, epithelial cells, dendritic cells, NK cells, smooth muscle cells, and proliferating cells (Figure 1B). Analysis of the cell‐type distribution between the sham‐operated (SHAM) and CLP groups showed that the proportion of endothelial cells was markedly increased in the CLP group (Figure 1C). Notably, *Pear1* expression was elevated in lung tissues from mice with CLP‐induced lung injury and was particularly increased in endothelial cells (Figure 1D–G). In addition, data from the Human Protein Atlas (HPA; http://www.proteinatlas.org) showed that PEAR1 is broadly expressed across human tissues and is highly expressed in the lung (Figure S1A).

To investigate whether PEAR1 participates in the pathological process of S‐ALI, we systematically evaluated its contribution to S‐ALI by establishing a CLP‐induced sepsis model in C57BL/6 mice according to standard methods [13], as illustrated in Figure 1H. Histological evaluation revealed that lung injury was significantly exacerbated at 18 h after the induction of sepsis (Figure S1B, S1C). The levels of lactate in the serum and lung tissue, together with the levels of the inflammatory mediators IL‐1β, IL‐6, and TNF‐α in the bronchoalveolar lavage fluid (BALF), peaked at 18 h after CLP (Figure S1D–F). Given that cell junctions are critical for maintaining vascular permeability, we also assessed the protein expression levels of ZO‐1, VE‐cadherin, Occludin, and Claudin‐1 and found that all the proteins were significantly downregulated at 18 h after CLP (Figure S1G). We quantified PEAR1 levels in the lung tissues of S‐ALI mice and found that *Pear1* mRNA and PEAR1 protein expression were significantly elevated (Figure 1I, J). Next, to further examine the expression and localization of PEAR1, we measured PEAR1 levels in the lung tissues of septic mice. Immunofluorescence (IF) double staining revealed that PEAR1 protein was primarily localized in the cytoplasm of ECs in lung tissues from S‐ALI model mice, and its expression was significantly higher than that in the control group (Figure 1K).

When mouse pulmonary microvascular endothelial cells (MPMVECs) were treated in vitro with 10 µg/mL LPS for 12 h, cell viability was not affected, whereas cell permeability was significantly increased (Figure S1H, I). Consistent with these observations, we found that LPS‐stimulated MPMVECs exhibited concentration‐ and time‐dependent reductions in the expression levels of ZO‐1, VE‐cadherin, Occludin, and Claudin‐1 proteins (Figure S1J, K). After MPMVECs were exposed to LPS (10 µg/mL, 12 h), the concentrations of inflammatory mediators and lactate in the cell supernatant increased significantly (Figure S1L, M). We further investigated the expression of PEAR1 in an in vitro model after LPS stimulation. LPS stimulation significantly increased *Pear1* mRNA expression and PEAR1 protein levels in MPMVECs (Figure 1L, M). The IF double staining results were consistent with the in vivo findings (Figure 1N).

Validation in clinical cohorts revealed elevated serum PEAR1 levels in S‐ARDS patients compared with those in healthy controls (HCs) (Figure 1O), and this increase was positively correlated with ARDS severity (Figure 1P). Furthermore, serum PEAR1 levels were significantly correlated with the oxygenation index, lactate level, Sequential Organ Failure Assessment (SOFA) score, Acute Physiology and Chronic Health Evaluation II (APACHE II) score, C‐reactive protein (CRP) level, and procalcitonin (PCT) level (Figure 1Q, R, Figure S1N–Q). Additional baseline data and clinical outcomes are shown in Table S1.

### 2.2 PEAR1 Promotes Pulmonary Vascular Hyperpermeability and Exacerbates ALI in Septic Mice

**FIGURE 2:**
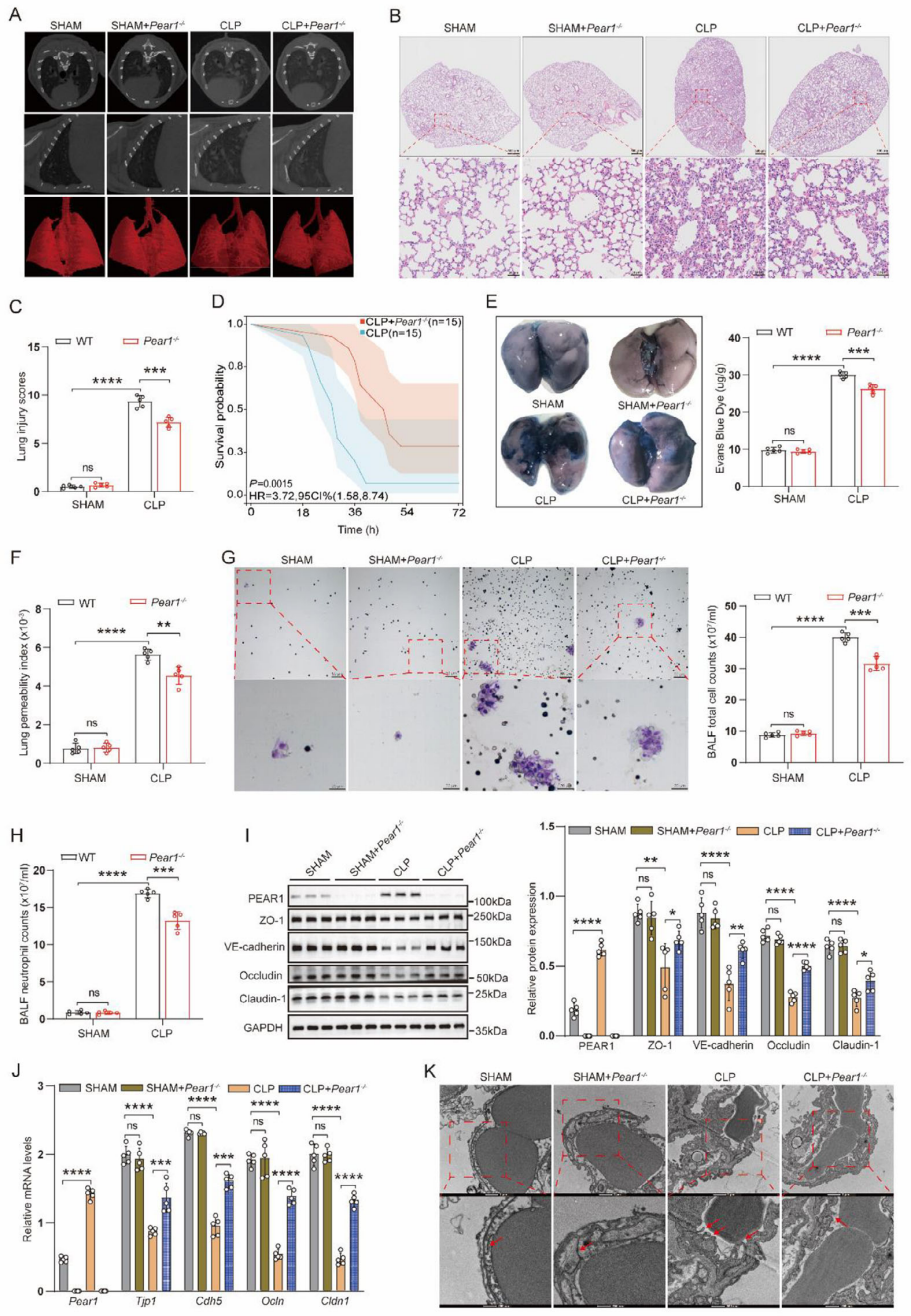
PEAR1 Promotes Pulmonary Vascular Hyperpermeability and Exacerbates ALI in Septic Mice. (A) Representative Micro‐CT and three‐dimensional (3D) reconstruction images from the lungs of WT and *Pear1* ^−/−^ mice after SHAM or CLP surgery. The defect sites represent exudative lesions and consolidation. (B, C). H&E staining of the lung tissue sections (B) and lung injury scores (C) in different groups (n = 5 per group). Scale bar: 500 µm (main) and 50 µm (inset). (D) Survival rates among CLP (n = 15) and CLP+ *Pear1* ^−/−^ (n = 15) mice were compared by Kaplan‐Meier test. (E) Evaluation of pulmonary vascular leakages using EBD extravasation assay. Leakage degrees were quantified by detecting the EBD contents in lung homogenate (n = 5 per group). (F) Calculation of the lung permeability index: protein content in BALF/protein content in plasma (n = 5 per group). (G) Modified Wright‐Giemsa staining of BALF precipitates, and quantification of total cells in BALF of each group (n = 5 per group). The red boxes show the neutrophils. Scale bar: 50 µm (main) and 20 µm (inset). (H) The counts of neutrophils in BALF of each group (n = 5 per group). (I) WB analysis of cell junction proteins (ZO‐1, VE‐cadherin, Occludin, and Claudin‐1) in lung tissue at 18 h in different groups (n = 5 per group). (J) RT‐qPCR analysis of cell junction protein mRNA levels in lung tissue at 18 h in different groups (n = 5 per group). (K) Representative TEM images showing cell junctions on the surface of pulmonary vessels in different groups (n = 5 per group). Scale bar: 1 µm (main) and 500 nm (inset). All data were represented as the means ± SD, *p < 0.05, **p < 0.01, ***p < 0.001, and ****p < 0.0001; ns, not significant.

To elucidate the underlying mechanisms through which PEAR1 mediates S‐ALI, polymicrobial sepsis was induced by CLP in *Pear1‐*deficient (*Pear1^−/−^*) and wild‐type (WT) mice. Samples of BALF, lung tissue, and blood were collected 18 h after the procedure for further examination. The genotyping results are shown in Figure S2A and B. PEAR1 expression in lung tissue was further validated by western blot (WB) analysis (Figure S2C). Micro‐CT imaging and histopathological analysis indicated that *Pear1* knockout alleviated lung injury in septic mice (Figure 2A–C). As expected, compared with WT mice in the CLP group, *Pear1* knockout improved survival of septic mice by 19.97% at 72 h (Figure 2D).

Endothelial dysfunction‐induced vascular permeability is a key driver of S‐ALI [5, 13]. To determine whether PEAR1 contributes to this process, we performed an Evans blue dye (EBD) extravasation assay and quantified dye leakage into lung tissue one hour after tail vein injection. Significant bilateral pulmonary vascular leakage, manifested as increased EBD extravasation into lung tissue, was observed in the CLP group. Genetic ablation of *Pear1* markedly reduced sepsis‐induced pulmonary vascular hyperpermeability, as quantified by the EBD assay (Figure 2E). These findings were complemented by a significant decrease in the pulmonary vascular permeability index (Figure 2F). To further characterize the inflammatory profile, we enumerated total cells and performed differential counts on Giemsa‐stained BALF smears at 18 h post‐CLP or SHAM surgery. Compared with SHAM surgery, CLP resulted in increased total cell and neutrophil counts in the BALF. However, these cell counts were significantly reduced following *Pear1* knockout (Figure 2G, H). We also evaluated the expression of ZO‐1, VE‐cadherin, Occludin, and Claudin‐1. The results showed that CLP intervention significantly decreased the protein levels of these molecules, whereas *Pear1* knockout significantly alleviated the CLP‐induced degradation of these proteins (Figure 2I). Moreover, the transcriptional levels of these molecules exhibited trends similar to their protein levels (Figure 2J). Consistent findings were further supported by transmission electron microscopy (TEM) (Figure 2K).

Furthermore, we constructed a vector carrying the *sh‐Pear1‐GFP* sequence delivered by adeno‐associated virus 9 (AAV9) and driven by the *Icam‐2* promoter to achieve endothelial‐specific knockdown. Fluorescence analysis indicated that AAV9 significantly targeted endothelial cells and effectively inhibited PEAR1 expression (Figure S2D,E). Endothelial‐specific knockdown of *Pear1* was confirmed to significantly alleviate sepsis‐induced lung injury and vascular hyperpermeability in mice, as assessed by hematoxylin and eosin (H&E) staining, EBD assay, and TEM (Figure S2F–I).

In subsequent in vitro experiments, CCK‐8 assays revealed that transfection with *Pear1*‐targeted siRNA had no effect on cell viability (Figure S2J). Consistent with the in vivo findings, LPS treatment (10 µg/mL, 12 h) significantly increased the permeability of MPMVECs. Following *Pear1*‐targeted siRNA intervention, MPMVEC permeability markedly reduced, as evidenced by elevated transendothelial electrical resistance (TEER) (Figure S2K). WB and reverse transcription quantitative polymerase chain reaction (RT‐qPCR) analyses were used to measure the protein and mRNA expression levels of cell junction molecules (ZO‐1, VE‐cadherin, Occludin, and Claudin‐1), respectively. As expected, LPS stimulation reduced the protein and mRNA expression levels of these cell junction molecules, whereas *Pear1* knockdown effectively mitigated these effects (Figure S2L,M). IF staining confirmed these findings (Figure S2N). Collectively, these results indicate that PEAR1 plays a significant biological role in sepsis‐induced pulmonary microvascular endothelial barrier dysfunction, leading to increased pulmonary vascular permeability and the promotion of ALI.

### 2.3 PEAR1 Regulates Glycolysis in PMVECs in a Model of S‐ALI

**FIGURE 3:**
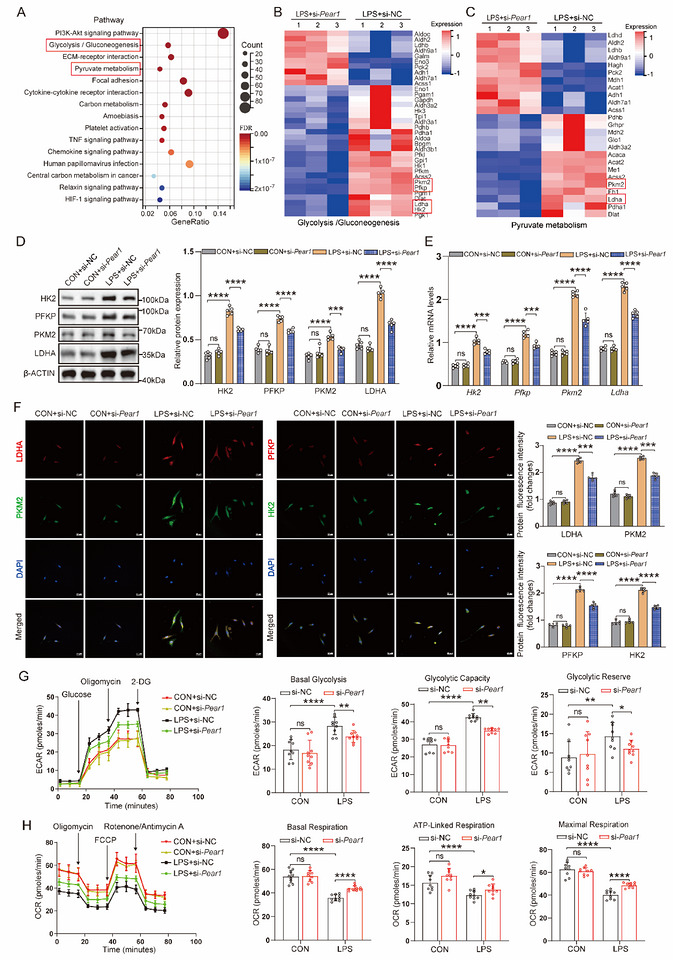
PEAR1 Regulates Glycolysis in PMVECs in a Model of S‐ALI. (A) Kyoto Encyclopedia of Genes and Genomes (KEGG) pathway analysis of differentially expressed genes (DEGs) from RNA‐seq of MPMVECs transfected with si‐NC or si‐*Pear1* and subsequent stimulation with LPS (10 µg/mL, 12 h) (n = 3 per group). (B, C) Heatmaps of DEGs in the glycolysis pathway and pyruvate metabolism from MPMVECs transfected with si‐NC or si‐*Pear1* and subsequent stimulation with LPS (n = 3 per group). (D) WB analysis of key glycolytic enzymes (HK2, PFKP, PKM2, and LDHA) in MPMVECs transfected with si‐NC or si‐*Pear1* and subsequent stimulation with or without LPS (n = 5 per group). (E) RT‐qPCR analysis of key glycolytic enzyme mRNA levels in MPMVECs in different groups (n = 5 per group). (F) Representative IF staining images and relative intensity quantification of LDHA (red), PKM2 (green), PFKP (red) and HK2 (green) in MPMVECs in different groups. Nuclei were stained with DAPI (blue) (n = 5 per group). Scale bar, 20 µm. (G) Extracellular acidification rate (ECAR) was measured in MPMVECs in absence of exogenous glucose, after sequential addition of 10 mM glucose followed by oligomycin (1 µM), and 2‐DG (50 mM). And basal glycolysis, glycolytic capacity, and glycolytic reserve were presented as bar graphs in different groups (n = 3 per group). (H) Oxygen consumption rate (OCR) was measured in MPMVECs during the sequential injection of oligomycin (1.5 µM), FCCP (1.5 µM), and a mixture of rotenone and antimycin A (0.5 µM). And basal respiration, ATP‐linked respiration, and Maximal respiration were presented as bar graphs in different groups (n = 3 per group). All data were represented as the means ± SD, *p < 0.05, **p < 0.01, ***p < 0.001, and ****p < 0.0001; ns, not significant.

To elucidate the mechanism by which PEAR1 contributes to S‐ALI, we conducted RNA‐seq analysis on LPS‐treated MPMVECs with or without genetic silencing of *Pear1*. RNA‐seq revealed that 709 genes were upregulated and 440 genes were downregulated in response to *Pear1* knockdown (|log2(FC)| > 1, *p* < 0.05) (Figure S3A). Subsequent KEGG enrichment analysis of the differentially expressed genes revealed significant alterations in glycolysis and pyruvate metabolism (Figure 3A). Consequently, we generated heatmaps of genes involved in the glycolysis pathway and pyruvate metabolism, which revealed several key glycolytic genes whose expression was attenuated in LPS‐stimulated *Pear1* knockdown MPMVECs (Figure 3B,C). Further targeted energy metabolomics analysis revealed that 35 metabolites, including glycolytic metabolites such as lactate, were decreased, whereas 15 metabolites were increased (|log2(FC)| > 0.5, *p* < 0.05) (Figure S3B,C). Consistent with the RNA‐seq data, WB analysis confirmed that the expression of key glycolytic enzymes, including HK2, PFKP, PKM2, and LDHA, was upregulated in LPS‐stimulated MPMVECs but downregulated in *Pear1*‐silenced MPMVECs (Figure 3D). Moreover, the mRNA expression levels of these molecules were consistent with their protein levels (Figure 3E). IF staining confirmed these findings (Figure 3F). Consistent results were observed in HPMECs following *PEAR1* knockdown, as determined by WB and RT‐qPCR analyses (Figure S3D,E). Taken together, our findings identify PEAR1 as an essential regulator of the glycolytic pathway in LPS‐stimulated PMVECs, as it modulates the expression of its key genes.

We further evaluated the glycolytic activity of MPMVECs in response to LPS stimulation to determine the influence of PEAR1 on metabolism. The extracellular acidification rate (ECAR) was used as an indicator of glycolytic function. LPS stimulation significantly increased glycolysis, the maximal glycolytic capacity, and the glycolytic reserve in endothelial cells, whereas *Pear1* knockdown reversed these changes (Figure 3G). We further evaluated mitochondrial oxidative phosphorylation in MPMVECs via oxygen consumption rate (OCR) assays. As shown in Figure 3H, LPS treatment markedly reduced mitochondrial respiration in MPMVECs. However, *Pear1* knockdown significantly restored the LPS‐induced decreases in basal respiration, maximal respiratory capacity, and reserve capacity in MPMVECs. Further assessment of lactate levels in the supernatants showed that both *Pear1* knockdown in MPMVECs and *PEAR1* knockdown in HPMECs led to a marked reduction in lactate levels (Figure S3F). Collectively, these metabolic data demonstrate that LPS stimulation enhances the glycolytic capacity of PMVECs. In the absence of PEAR1, PMVECs partially shifted their energy metabolism from glycolysis to oxidative phosphorylation.

We investigated the specific role of PEAR1 in vivo by generating *Pear1* ^−^/^−^ mice. WB analysis revealed that *Pear1* deficiency downregulated the expression of the key glycolytic enzymes HK2, PFKP, PKM2, and LDHA in the lung tissue of CLP‐induced S‐ALI mice (Figure S3G,H), which was confirmed by IF staining (Figure S3I–K). Consistently, *Pear1* knockout significantly reduced lactate levels in the lung tissues (Figure S3L). Collectively, these data indicate that LPS triggers PEAR1‐mediated metabolic reprogramming characterized by increased glycolysis in PMVECs.

### 2.4 PEAR1 Increases HIF‐1α Binding to Importin α via K172 Lactylation to Promote HIF‐1α Nuclear Localization

**FIGURE 4:**
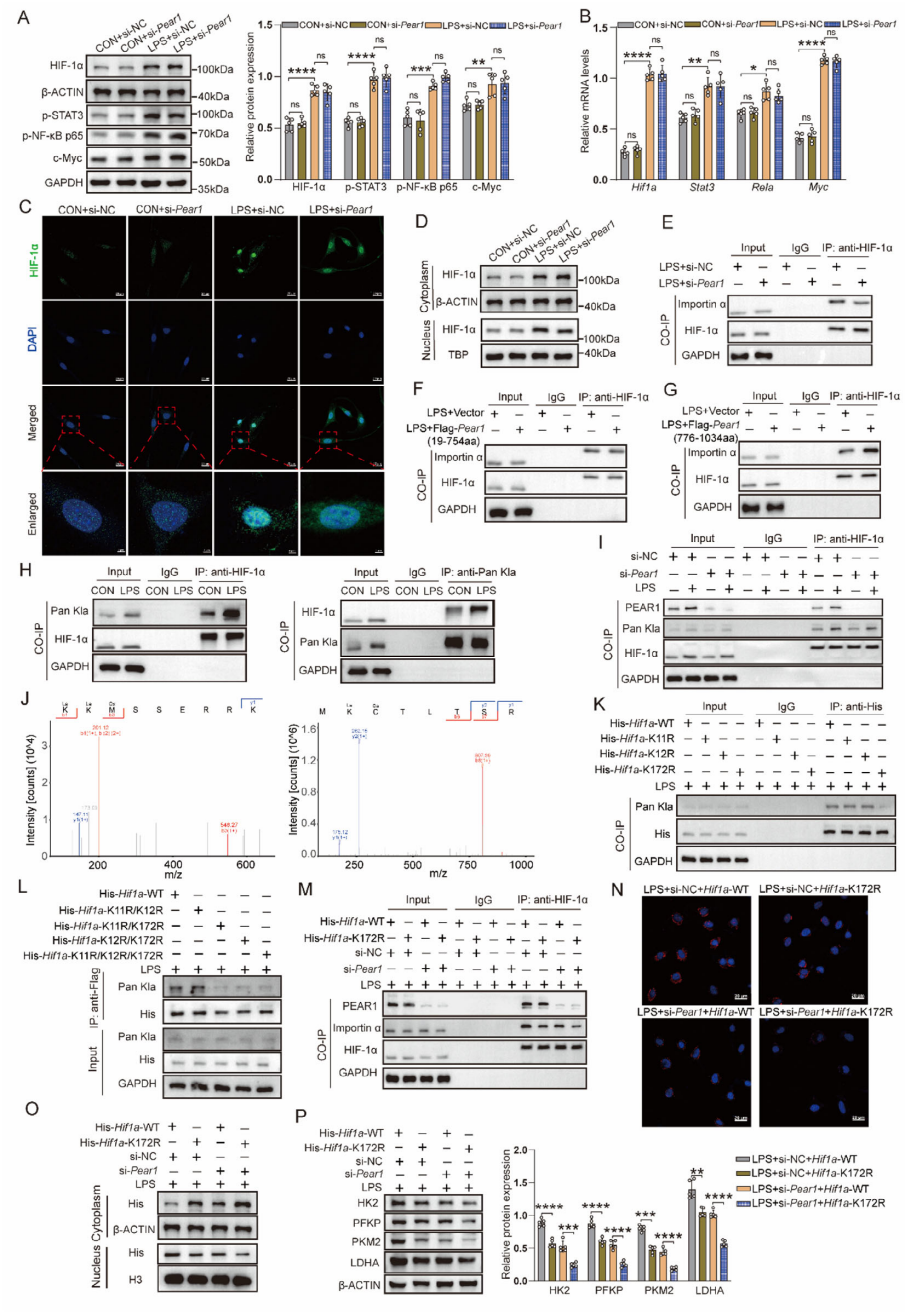
PEAR1 Increases HIF‐1α Binding to Importin α via K172 Lactylation to Promote HIF‐1α Nuclear Localization. (A) WB analysis of HIF‐1α, c‐Myc, p‐STAT3, and p‐NF‐κB p65 in MPMVECs transfected with si‐NC or si‐*Pear1* and subsequent stimulation with or without LPS (n = 5 per group). (B) RT‐qPCR analysis of *Hif1a*, *Myc, Stat3*, and *Rela* mRNA levels in MPMVECs in different groups (n = 5 per group). (C) Representative IF staining images of HIF‐1α (green) in MPMVECs in different groups. And nuclei were stained with DAPI (blue). Scale bar: 20 µm (main) and 4 µm (inset). (D) WB analysis of cytoplasmic and nuclear HIF‐1α expression in MPMVECs in different groups (n = 5 per group). (E) Co‐immunoprecipitation (Co‐IP) was performed to examine the interaction between HIF‐1α and importin α in MPMVECs transfected with si‐NC or si‐*Pear1* and subsequent stimulation with LPS (10 µg/mL for 12 h). (F) Co‐IP was performed to examine the interaction between HIF‐1α and importin α in LPS‐stimulated MPMVECs with or without overexpressing *Pear1* (19‐754 aa). (G) Co‐IP was performed to examine the interaction between HIF‐1α and importin α in LPS‐stimulated MPMVECs with or without overexpressing *Pear1* (776‐1034 aa). (H) Co‐IP was performed to examine lactylation of HIF‐1α in MPMVECs after treatment with or without LPS (10 µg/mL for 12 h). (I) Co‐IP was performed to examine lactylation of HIF‐1α in MPMVECs transfected with si‐NC or si‐*Pear1* and subsequent stimulation with or without LPS (10 µg/mL for 12 h). (J) Illustration of possible lactylation sites of HIF‐1α in the MPMVECs analyzed via IP‐LC‐MS/MS. Three possible lactylation sites of HIF‐1α observed are shown. (K, L) MPMVECs were transfected with His‐*Hif1a*‐WT or single‐site mutant (K11R, K12R, and K172R) (K) multi‐site mutant plasmids (K11R/K12R, K11R/K172R, K12R/K172R, and K11R/K12R/K172R) (L). Immunoprecipitation of exogenously expressed HIF‐1α was performed using an anti‐His antibody, and lactylation levels were assessed with an anti‐Pan‐Kla antibody. (M) Co‐IP was performed to examine the interaction between HIF‐1α and importin α after overexpressed His‐*Hif1a*‐WT or His‐*Hif1a*‐K172R in LPS‐stimulated MPMVECs with or without *Pear1* silencing. (N) Interaction between HIF‐1α and importin α detected by PLA experiments in LPS‐stimulated MPMVECs in different groups. Red fluorescent puncta indicate PLA signals, representing interaction or close proximity between HIF‐1α and importin α. Nuclei were stained with DAPI (blue). Scale bar, 20 µm. (O) WB analysis of cytoplasmic and nuclear HIF‐1α expression in LPS‐stimulated MPMVECs in different groups. (P) WB analysis of HK2, PFKP, PKM2, and LDHA in LPS‐stimulated MPMVECs in different groups (n = 5 per group). All data were represented as the means ± SD, *p < 0.05, **p < 0.01, ***p < 0.001, and ****p < 0.0001; ns, not significant.

To explore the specific mechanism by which PEAR1 regulates the key enzymes involved in glycolysis in MPMVECs, we predicted the common upstream transcription factors of HK2, PFKP, PKM2, and LDHA using the PROMO database (Table S4). By taking the intersection of these predictions and integrating evidence from the literature, we identified HIF‐1α, c‐Myc, STAT3 and NF‐κB p65 [26, 27, 28, 29] (Figure S4A). As shown in Figure 4A,B, in MPMVECs, *Pear1* knockdown did not affect the expression of the above transcription factors, either at the protein activation level or at the transcriptional level. However, IF staining revealed that HIF‐1α was predominantly located in the nucleus upon LPS stimulation, and this process was facilitated by PEAR1 (Figure 4C). No significant changes in the levels of c‐MYC, phospho‐STAT3, or phospho‐NF‐κB p65 were observed (Figure S4B). Subcellular fractionation demonstrated a reciprocal effect of PEAR1 on HIF‐1α localization: HIF‐1α nuclear translocation was reduced following *Pear1* knockdown (Figure 4D). IF staining of HPMECs showed results consistent with the above observations (Figure S4C).

The nuclear import of transcription factors (TFs) is mediated by their nuclear localization signal (NLS). This signal is bound by importins, cytoplasmic carrier proteins that escort the TFs through the nuclear pores. Usually, NLSs are composed of continuous basic amino acid residues [30, 31]. In addition, given that importin α has been reported to mediate HIF‐1α nuclear import through nuclear localization signal‐dependent binding [32, 33], we next examined whether PEAR1 affects the interaction between HIF‐1α and importin α. Coimmunoprecipitation (co‐IP) assays showed that *Pear1* silencing significantly inhibited the interaction between HIF‐1α and importin α in LPS‐stimulated MPMVECs (Figure 4E). Structural prediction using AlphaFold revealed that PEAR1 is a transmembrane protein comprising an extracellular domain (19‐754 aa) and an intracellular domain (776‐1034 aa) (Figure S4D). We independently overexpressed its intracellular and extracellular domains and observed that overexpression of the intracellular domain significantly enhanced the binding between HIF‐1α and importin α, whereas overexpression of the extracellular domain had no effect. These findings indicate that the intracellular domain of PEAR1 is critical for regulating the nuclear localization of HIF‐1α (Figure 4F,G).

The nuclear translocation of TFs is strictly regulated after their biosynthesis and processing. Some PTMs, such as SUMOylation and lactylation, play a role in altering the subcellular localization of modified proteins [34, 35]. Therefore, we attempted to determine whether HIF‐1α can regulate its subcellular localization through lactylation. MPMVECs were treated with or without LPS, followed by co‐IP with anti‐HIF‐1α and anti‐Pan‐lysine lactylation (Kla) antibodies. The results revealed that HIF‐1α undergoes lactylation, which is increased upon LPS treatment (Figure 4H). To determine whether PEAR1 regulates HIF‐1α lactylation, we performed co‐IP. Both *Pear1* knockdown in MPMVECs and *PEAR1* knockdown in HPMECs decreased HIF‐1α lactylation (Figure 4I, Figure S4E). To identify the specific lactylation sites of HIF‐1α in LPS‐induced MPMVECs, we performed LC‐MS/MS analysis, which identified residues K11, K12 and K172 as possible lactylation sites (Figure 4J). We transfected MPMVECs with plasmids containing His‐tagged wild‐type *Hif1a*, single‐site mutants (K11R, K12R, and K172R), and multi‐site mutants (K11R/K12R, K11R/K172R, K12R/K172R, and K11R/K12R/K172R) to assess their effects on HIF‐1α lactylation. We performed co‐IP of exogenously expressed HIF‐1α using an anti‐His antibody and assessed lactylation levels using an anti‐Pan‐Kla antibody. The results indicated that the mutants containing K172R significantly reduced HIF‐1α lactylation, whereas the K11R and K12R mutants had no significant effect (Figure 4K, L). We further transfected *HIF1A*‐WT and *HIF1A*‐K172R into HPMECs, and the results again showed that the K172R mutation significantly inhibited HIF‐1α lactylation (Figure S4F). These findings identify K172 as the principal lactylation site in HIF‐1α. As shown in Figure S4G, K172 of HIF‐1α is relatively conserved among species. To determine whether increased K172la in HIF‐1α promotes its nuclear localization, we separately overexpressed *Hif1a*‐WT and *Hif1a*‐K172R in MPMVECs. Compared with WT HIF‐1α, the K172R mutation strongly reduced the binding of HIF‐1α to importin α, resulting in decreased nuclear import (Figure 4M,O). Proximity Ligation Assay (PLA) experiments revealed that the K172R mutation caused a reduction in the binding between HIF‐1α and importin α (Figure 4N). Furthermore, as shown in Figure 4P, Figure S4H–K, glycolysis and lactate levels were significantly reduced after the K172R mutation of HIF‐1α. Similar results were obtained in HPMECs (Figure S4I–K). Collectively, these results demonstrate that PEAR1 promotes the binding of HIF‐1α to importin α driven by increased HIF‐1α K172 lactylation, which facilitates the nuclear translocation of HIF‐1α and subsequent glycolysis.

### 2.5 PEAR1 Mediates the Binding of AARS1 to HIF‐1α, Thereby Regulating HIF‐1α Lactylation

**FIGURE 5:**
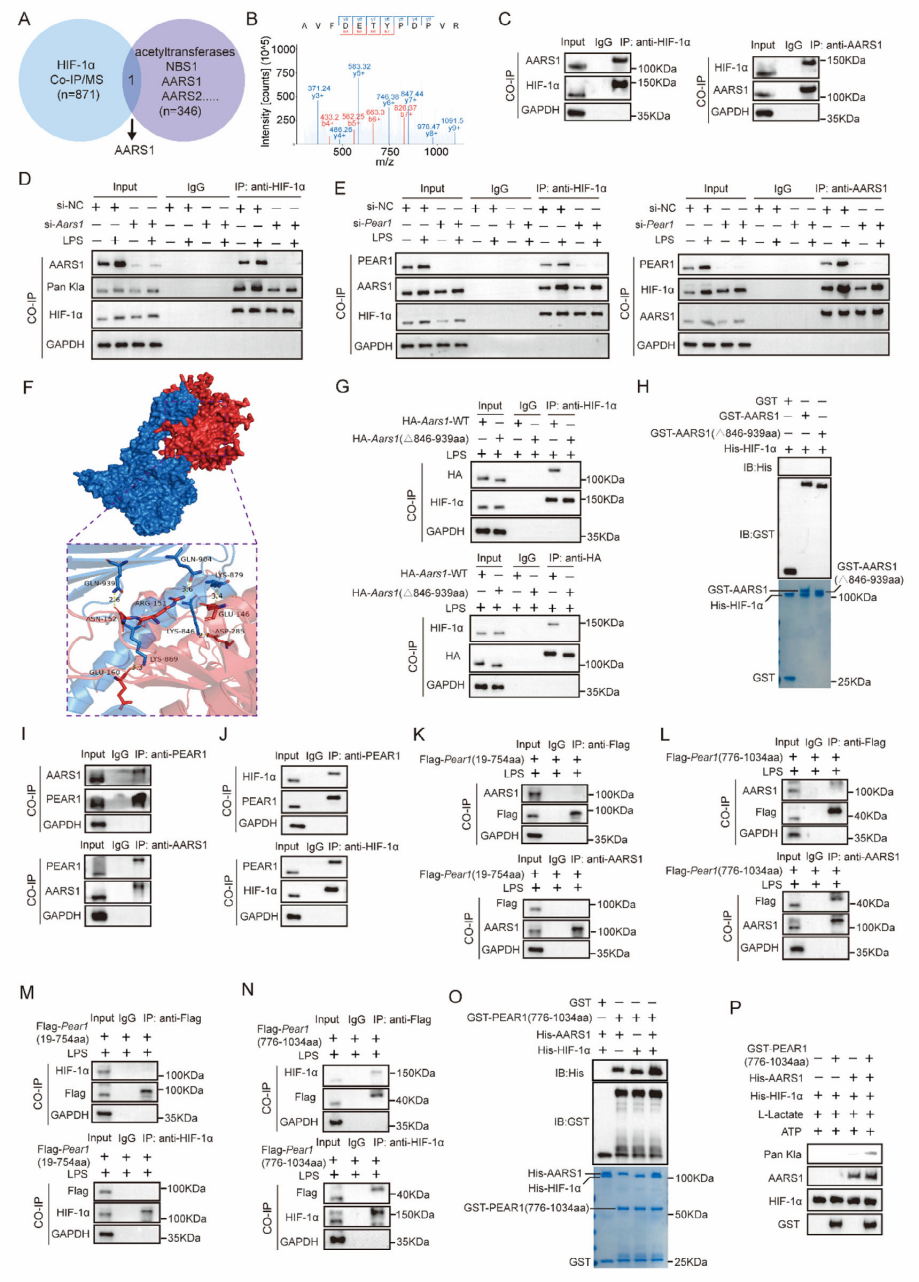
PEAR1 Mediates the Binding of AARS1 to HIF‐1α, thereby Regulating HIF‐1α Lactylation. (A) Identifying HIF‐1α­interacting lactyltransferase using co‐IP in combination with LC‐MS/MS. (B) The spectrograms showed mass spectroscopy‐identified AARS1 peptides. (C) Co‐IP was performed to examine the interaction between HIF‐1α and AARS1 in MPMVECs treated with LPS (10 µg/mL for 12 h). (D) Co‐IP was performed to examine lactylation of HIF‐1α in MPMVECs transfected with si‐NC or *Aars1*‐targeted small interfering RNA (si‐*Aars1*) and subsequent stimulation with or without LPS (10 µg/mL for 12 h). (E) Co‐IP was performed to examine the interaction between AARS1 and HIF‐1α in MPMVECs transfected with si‐NC or si‐*Pear1* and subsequent stimulation with or without LPS (10 µg/mL for 12 h). (F) Molecular docking model of HIF‐1α (red) (PDB entry Q61221) interacting with AARS1 (PDB entry Q8BGQ7) (blue). (G) Co‐IP was performed to examine the interaction between HIF‐1α and exogenously expressed proteins from the overexpressed plasmid HA‐*Aars1* or the truncated plasmid HA‐*Aars1* (Δ846‐939 aa), in MPMVECs treated with LPS (10 µg/mL for 12 h). (H) GST‐pull down assay was performed to examine the direct interaction between AARS1‐WT or AARS1 (Δ846‐939 aa) with HIF‐1α, respectively. (I) Co‐IP was performed to examine the interaction between AARS1 and PEAR1 in LPS‐stimulated MPMVECs. (J) Co‐IP was performed to examine the interaction between HIF‐1α and PEAR1 in LPS‐stimulated MPMVECs. (K) Co‐IP was performed to examine the interaction between AARS1 and PEAR1 extracellular domain (19‐754 aa) in LPS‐stimulated MPMVECs. (L) Co‐IP was performed to examine the interaction between AARS1 and PEAR1 intracellular domain (776‐1034 aa) in LPS‐stimulated MPMVECs. (M) Co‐IP was performed to examine the interaction between HIF‐1α and PEAR1 extracellular domain (19‐754 aa) in LPS‐stimulated MPMVECs. (N) Co‐IP was performed to examine the interaction between HIF‐1α and PEAR1 intracellular domain (776‐1034 aa) in LPS‐stimulated MPMVECs. (O) GST‐pull down assay was performed to examine the direct interaction between HIF‐1α, AARS1 and PEAR1 intracellular domain (776‐1034 aa) in LPS‐stimulated MPMVECs. (P) In vitro HIF‐1α lactylation assay: Purified HIF‐1α was co‐incubated with the purified intracellular domain of PEAR1 (776‐1034 aa), purified AARS1 and L‐lactate. The lactylation level of HIF‐1α was analyzed using the anti‐Pan Kla antibody.

Considering that the enzymatic machinery responsible for lysine lactylation remains incompletely defined and partially overlaps with canonical lysine acetyltransferases, we constructed a curated candidate acyltransferase/lactylation‐related enzyme panel, which included annotated acetyltransferases and recently reported lactylation writers and regulators [25, 36, 37, 38]. This panel was then intersected with the HIF‐1α‐associated proteins identified by co‐IP/MS. Among the candidates meeting our screening criteria, alanyl‐tRNA synthetase 1 (AARS1) was the only enzyme identified and was therefore prioritized for further validation (Figure 5A,B). Subsequent co‐IP analysis further demonstrated the interaction between HIF‐1α and AARS1 (Figure 5C). The AARS1 functions as both a lactate sensor and a lactate transferase [38, 39]. We then proceeded to knockdown or overexpress *Aars1* to investigate how AARS1 affects HIF‐1α lactylation. Co‐IP revealed that *Aars1* knockdown significantly reduced the lactylation level of HIF‐1α (Figure 5D), whereas *Aars1* overexpression markedly increased its lactylation level (Figure S5A). Furthermore, we discovered that in MPMVECs, *Pear1* knockdown markedly reduced AARS1 binding to HIF‐1α (Figure 5E), whereas *Pear1* overexpression enhanced this interaction (Figure S5B,C). Similar results were obtained in HPMECs following *PEAR1* knockdown (Figure S5D).

Molecular docking revealed the binding site of HIF‐1α on AARS1 (Figure 5F). To define the catalytically active region of AARS1, we generated HA‐tagged versions of the full‐length protein and a C‐terminal truncation mutant (Δ846‐939 aa) on the basis of its known structural domains. Co‐IP revealed that compared with full‐length AARS1, deletion of this domain (846‐939 aa) prevented its interaction with HIF‐1α (Figure 5G). However, the results of the GST pull‐down assays revealed that the GST‐tagged full‐length AARS1 protein and the AARS1 truncation mutants failed to directly bind to HIF‐1α (Figure 5H). We therefore hypothesized that PEAR1 might act as a bridging protein in this interaction. Co‐IP confirmed that PEAR1 binds to both AARS1 and HIF‐1α (Figure 5I,J), and this interaction is mediated by the PEAR1 intracellular domain (776‐1034 aa) (Figure 5K–N). To investigate the specific binding sites of PEAR1 with AARS1 and HIF‐1α, we conducted molecular docking prediction analysis. The results showed that the PEAR1 residues GLY‐1014, HIS‐1015, PRO‐1019, and ARG‐1022 formed hydrogen bonds with the AARS1 residues ASP‐180, THR‐38, TYR‐322, ASP‐416, and GLY‐419, with a binding energy of ‐202.40 kcal/mol (Figure S5E). PEAR1 residues ARG‐1022, VAL‐1021, HIS‐1023, TYR‐1016, and HIS‐1011 formed hydrogen bonds with HIF‐1α residues GLY‐76, LEU‐793, GLN‐795, PHE‐91, and GLN‐87, respectively, with a binding energy of ‐210.32 kcal/mol (Figure S5F). Therefore, we constructed a truncation mutant lacking the PEAR1 1011–1023 aa region. Co‐IP revealed that deletion of this region significantly inhibited the binding of PEAR1 to AARS1 and HIF‐1α (Figure S5G,H). We further mutated the PEAR1 binding sites for AARS1 and HIF‐1α to alanine. Co‐IP revealed that mutation at position 1022 of PEAR1 significantly inhibited its binding to both AARS1 and HIF‐1α (Figure S5I, J). These findings suggest that the 1022 residue of PEAR1 may be a key site promoting HIF‐1α lactylation and nuclear translocation. Further validation via GST pull‐down assays revealed that the intracellular segment (776‐1034 aa) of the purified PEAR1 protein binds to both AARS1 and HIF‐1α, with all three components capable of forming a ternary complex (Figure 5O). Furthermore, an in vitro lactylation assay demonstrated that the purified intracellular segment (776‐1034 aa) of PEAR1 promotes the interaction between AARS1 and HIF‐1α and regulates HIF‐1α lactylation (Figure 5P). These findings indicate that PEAR1 mediates AARS1 binding to HIF‐1α via its intracellular segment, which in turn promotes HIF‐1α lactylation.

### 2.6 Glycolysis‐Derived Lactate Promotes PEAR1 Expression Through H3K18la During S‐ALI

**FIGURE 6:**
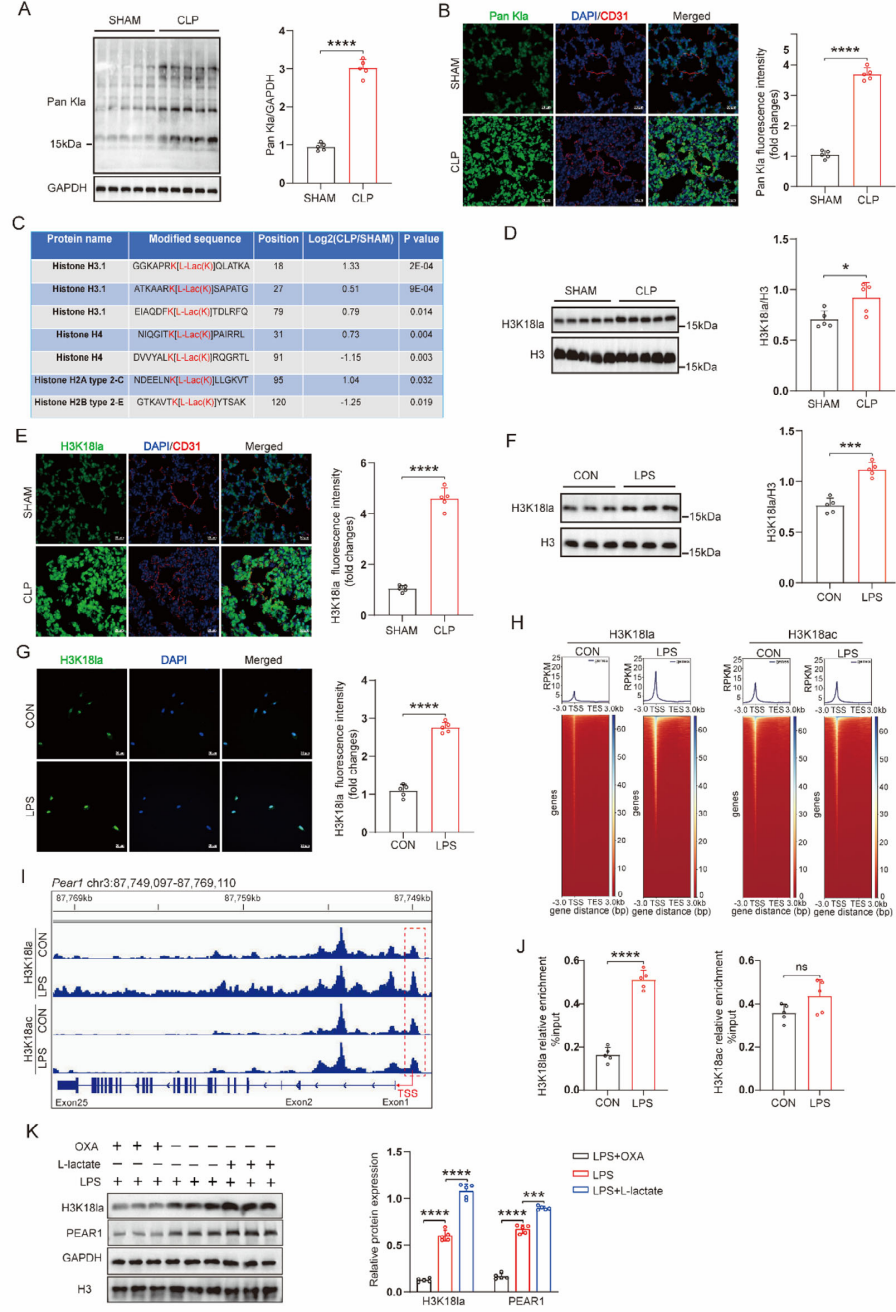
Glycolysis‐Derived Lactate Promotes PEAR1 Expression through H3K18la during S‐ALI. (A) WB analysis of Pan‐lysine lactylation (Kla) protein levels in lung tissues from SHAM and CLP groups (n = 5 per group). (B) Representative IF staining images and relative intensity quantification of Pan‐Kla (green) in lung tissues from SHAM and CLP groups (n = 5 per group). Vascular endothelium was labeled with CD31 (red), and nuclei were stained with DAPI (blue). Scale bar, 20 µm. (C) Proteomic lactylation analysis revealed significantly altered histone lactylation sites in lung tissue from SHAM and CLP groups (n = 3 per group). (D) WB analysis of H3K18la protein levels in lung tissues from SHAM and CLP groups (n = 5 per group). (E) Representative IF staining images and relative intensity quantification of H3K18la (green) in lung tissues from SHAM and CLP groups (n = 5 per group). Vascular endothelium was labeled with CD31 (red), and nuclei were stained with DAPI (blue). Scale bar, 20 µm. (F) WB analysis of H3K18la protein levels in MPMVECs with or without LPS stimulation (10 µg/mL for 12 h) (n = 5 per group). (G) Representative IF staining images and relative intensity quantification of H3K18la (green) in MPMVECs with or without LPS stimulation (10 µg/mL for 12 h) (n = 5 per group). Nuclei were stained with DAPI (blue). Scale bar, 20 µm. (H) Heat maps of the genome occupancy of H3K18la and H3K18ac ± 3 kb flanking transcription start sites in MPMVECs with or without LPS stimulation (10 µg/mL for 12 h) from CUT&Tag analysis (n = 3 per group). (I) IGV tracks for *Pear1* from CUT&Tag analysis in MPMVECs. (J) ChIP‐qPCR assays of H3K18la and H3K18ac occupancy rates in the promoter region of *Pear1* in MPMVECs with or without LPS stimulation (10 µg/mL for 12 h) (n = 5 per group). (K) MPMVECs were pre‐treated with sodium oxamate (OXA) (20 mM for 3 h), lactate (8 mM for 7 h) or PBS, followed stimulated by LPS (10 µg/mL for 12 h). H3K18la and PEAR1 protein levels were detected by WB analysis (n = 5 per group). All data were represented as the means ± SD, *p < 0.05, **p < 0.01, ***p < 0.001, and ****p < 0.0001; ns, not significant.

Because HIF‐1α K172 lactylation promoted HIF‐1α nuclear translocation and subsequently enhanced glycolysis and lactate production, we next investigated whether the accumulated lactate could feed back to regulate PEAR1 expression, thereby sustaining the PEAR1‐AARS1‐HIF‐1α axis. Given that lactate‐derived histone lactylation has been shown to promote gene transcription and participate in endothelial injury during S‐ALI, as demonstrated in our previous study, we hypothesized that glycolysis‐derived lactate may induce *Pear1* transcription through histone lactylation [25, 40]. To test this hypothesis, we first assessed global lactylation levels and observed elevated global lactylation in CLP‐induced septic mouse lung tissues (Figure 6A,B), with proteomic lactylation analysis identifying H3K18la as the most prominently altered histone modification site (Figure 6C). WB analyses validated these findings (Figure 6D, Figure S6A). IF staining also showed consistent results (Figure 6E). Similarly, in vitro experiments involving LPS‐stimulated MPMVECs and HPMECs also showed a significant increase in H3K18la levels (Figure 6F,G and Figure S6B,C).

We then investigated whether H3K18la directly participates in the transcriptional regulation of *Pear1*. CUT&Tag analysis revealed that LPS stimulation distinctly modified the genomic occupancy of H3K18la in MPMVECs, whereas the binding pattern of H3K18ac remained largely unaltered (Figure 6H). Additionally, H3K18la enrichment in the promoter region was markedly increased in MPMVECs following LPS stimulation (Figure S6D). Similarly, CUT&Tag analysis in HPMECs revealed that LPS stimulation distinctly modified the genomic occupancy of H3K18la (Figure S6E). Further analysis using the Integrative Genomics Viewer (IGV) showed that LPS stimulation led to a significant increase in H3K18la enrichment, but not in H3K18ac enrichment, at the *Pear1* promoter region in MPMVECs (Figure 6I). In HPMECs, IGV analysis also confirmed a marked increase in H3K18la enrichment at the *PEAR1* promoter following LPS stimulation (Figure S6F). We subsequently verified the CUT&Tag results using chromatin immunoprecipitation (ChIP)‐qPCR, confirming that H3K18la is enriched at the *Pear1*/*PEAR1* promoter region (Figure 6J, Figure S6G). To determine whether this process was dependent on lactate production, we inhibited glycolysis‐derived lactate generation using sodium oxamate (OXA). OXA treatment markedly reduced H3K18la levels and suppressed PEAR1 expression, whereas supplementation with exogenous lactate restored or further increased both H3K18la and PEAR1 levels (Figure 6K). These findings indicate that glycolysis‐derived lactate promotes PEAR1 expression through H3K18la enrichment at its promoter. Together, these results define H3K18la as the epigenetic component that links HIF‐1α‐driven glycolysis and lactate accumulation back to *Pear1*/*PEAR1* transcription, which further reinforces the positive feedback loop of PEAR1‐AARS1‐HIF‐1α K172la‐glycolysis.

### 2.7 E‐selectin Binding Peptide‐Modified Liposomes Carrying *Pear1*‐Targeted Small Interfering RNA Ameliorate ALI in Mice With Polymicrobial Sepsis

**FIGURE 7:**
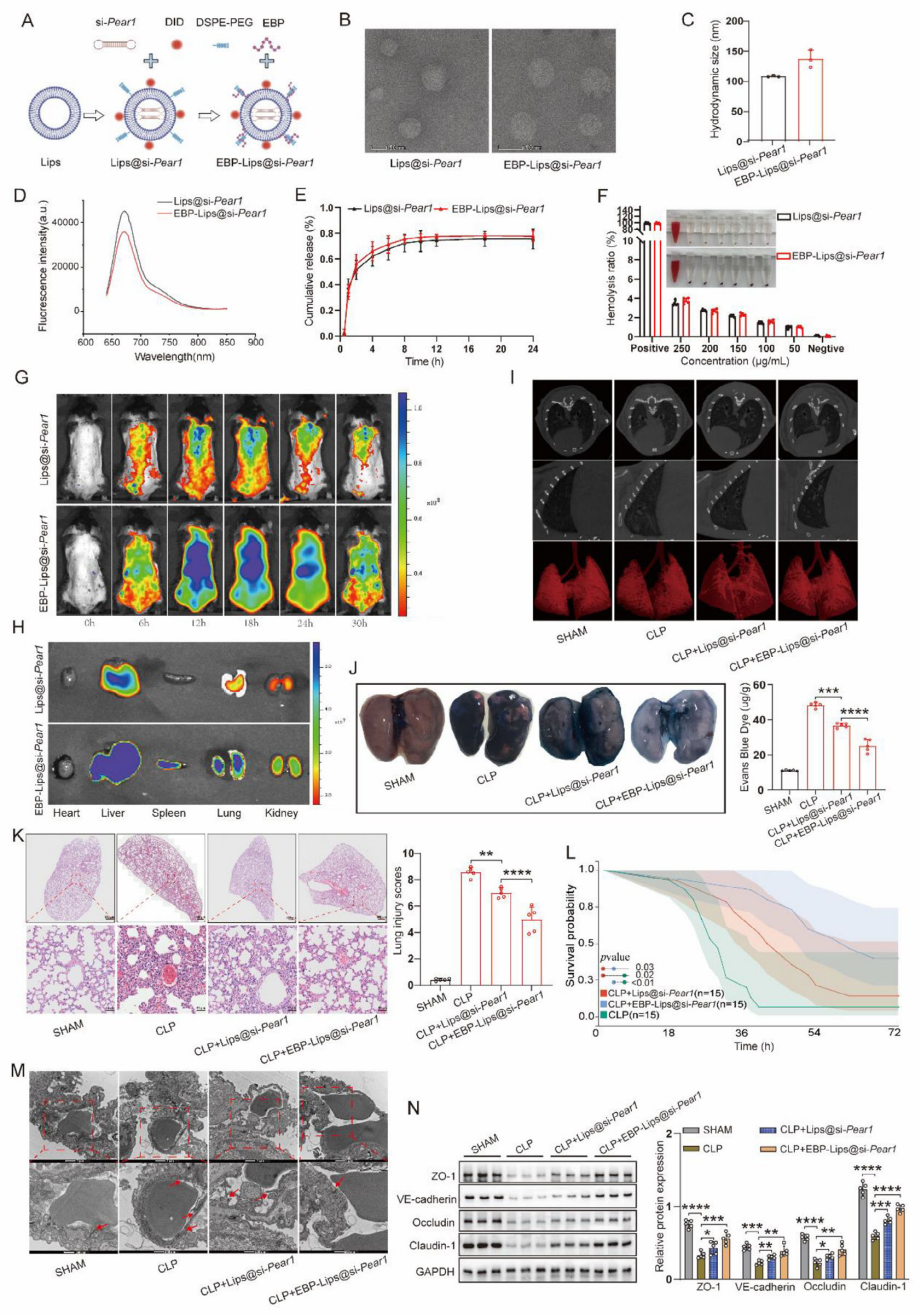
E‐selectin Binding Peptide‐Modified Liposomes Carrying *Pear1*‐Targeted Small Interfering RNA Ameliorate ALI in Mice with Polymicrobial Sepsis. (A) Schematic diagram of the synthesis for two nanomaterials (E‐selectin binding peptide‐modified liposomes carrying *Pear1*‐targeted small interfering RNA (EBP‐Lips@si‐*Pear1*) and unmodified liposomes carrying *Pear1*‐targeted small interfering RNA (Lips@si‐*Pear1*)). (B) Representative TEM images of Lips@si‐*Pear1* and EBP‐Lips@si‐*Pear1* (n = 5 per group). Scale bar,50 nm. (C) Dynamic light scattering (DLS) was performed on Lips@si‐*Pear1* and EBP‐Lips@si‐*Pear1* in aqueous solution (n = 3 per group). (D) The fluorescence intensities of Lips@si‐*Pear1* and EBP‐Lips@si‐*Pear1* (n = 3 per group). (E) The drug release rate curves of Lips@si‐*Pear1* and EBP‐Lips@si‐*Pear1* (n = 5 per group). (F) The hemolytic assessments of Lips@si‐*Pear1* and EBP‐Lips@si‐*Pear1* were conducted at concentration range of 50 to 250 µg/mL (n = 5 per group). (G) Fluorescence imaging of CLP mice at different time points after administration of Lips@si‐*Pear1* or EBP‐Lips@si‐*Pear1*. (H) Fluorescence imaging of CLP mice organs at 12 h after administration of Lips@si‐*Pear1* or EBP‐Lips@si‐*Pear1*. (I) Representative Micro‐CT and three‐dimensional (3D) reconstruction images of mouse lungs from SHAM, CLP, CLP + Lips@si‐*Pear1*, and CLP + EBP‐Lips@si‐*Pear1* groups. (J) Pulmonary vascular leakage in different groups of mice was assessed by EBD extravasation assay (n = 5 per group). (K) H&E staining of the lung tissue sections and injury scores in different groups (n = 5 per group). Scale bar: 500 µm (main) and 50 µm (inset). (L) Survival rates among CLP (n = 15), CLP + EBP‐Lips@si‐*Pear1* (n = 15) and CLP + Lips@si‐*Pear1* (n = 15) mice were compared by Kaplan‐Meier test. (M) Representative TEM images showing cell junctions on the surface of pulmonary vessels in different groups (n = 5 per group). Scale bar: 1 µm (main) and 500 nm (inset). (N) WB analysis of cell junction proteins (ZO‐1, VE‐cadherin, Occludin, and Claudin‐1) in lung tissue in different groups (n = 5 per group). All data were represented as the means ± SD, *p < 0.05, **p < 0.01, ***p < 0.001, and ****p < 0.0001; ns, not significant.

To further verify the role of PEAR1 in acute lung injury and microvascular permeability in septic mice, we developed E‐selectin binding peptide‐modified liposomes carrying *Pear1*‐targeted small interfering RNA (EBP‐Lips@si‐*Pear1*). The drug loading rate and encapsulation efficiency were 3.9% and 92.68%, respectively, which were not significantly different from those of the unmodified liposomes carrying *Pear1*‐targeted small interfering RNA (Lips@si‐*Pear1*) (the drug loading rate and encapsulation efficiency were 3.8% and 92.52%, respectively) (Figure 7A). TEM revealed that Lips@si‐*Pear1* and EBP‐Lips@si‐*Pear1* were spherical and well dispersed (Figure 7B). Dynamic light scattering (DLS) measurements in aqueous solution revealed sizes of 106.67 ± 3.95 and 137.15 ± 14.70 nm, respectively (Figure 7C). The polydispersity indices (PDIs) were 0.125 ± 0.010 and 0.145 ± 0.012, respectively (Figure S7A), and the zeta potential was 30.71 ± 2.59 mV for Lips@si‐*Pear1* and 30.15 ± 5.92 mV for EBP‐Lips@si‐*Pear1* (Figure S7B). Both Lips@si‐*Pear1* and EBP‐Lips@si‐*Pear1* exhibited the strongest fluorescence intensity at 675 nm (Figure 7D). The maximum drug release rate from EBP‐Lips@si‐*Pear1* was 73.88% after 12 h, and that from Lips@si‐*Pear1* was 72.16% after 18 h (Figure 7E). The hemolysis of both types of liposomes was evaluated at concentrations ranging from 50 to 250 µg/mL. With increasing Lips@si‐*Pear1* and EBP‐Lips@si‐*Pear1* concentrations, hemolysis slowly increased. The results revealed that the hemolysis rate of the liposomes was less than 4% at 250 µg/mL, and the toxicity was negligible (Figure 7F). CCK‐8 assays revealed that the activity of 200 µg/mL EBP‐Lips@si‐*Pear1* decreased significantly after 6 h of treatment with MPMVECs (Figure S7C).

Therefore, in our next experiment, the concentration of EBP‐Lips@si‐*Pear1* used to treat MPMVECs was 150 µg/mL. IF staining revealed significant cellular uptake of the EBP‐Lips@si‐*Pear1* at 12 h and significant inhibition of PEAR1 expression (Figure S7D). We then determined the in vivo doses of EBP‐Lips@si‐*Pear1* and Lips@si‐*Pear1* based on the relevant literature and the encapsulation efficiency of the nanoliposomes, and injected them intravenously at 13 mg/kg for subsequent studies [41]. Histopathological examination revealed that EBP‐Lips@si‐*Pear1* caused no significant pathological damage to the heart, liver, spleen, lungs, or kidneys (Figure S7E). In vivo fluorescence imaging of the mice and organs revealed significant enrichment of EBP‐Lips@si‐*Pear1* in organs such as the lung, liver, spleen and kidney at 12 h, whereas the targeting ability of Lips@si‐*Pear1* significantly reduced (Figure 7G,H). Furthermore, the lung tissue was lysed into single‐cell suspensions, and flow cytometry experiments were conducted. The results demonstrated that the colocalization of EBP‐modified liposomes (EBP‐Lips@si‐*Pear1*) with CD31^+^ endothelial cells was markedly greater (71.6%) (Figure S7F) than it was CD326^+^ epithelial cells (14.6%) (Figure S7G) and F4/80^+^ macrophages (23.1%) (Figure S7H). In contrast, non‐modified liposomes (Lips@si‐*Pear1*) did not clearly co‐localize with any of these three cell populations (Figure S7I–K).

To further examine the efficacy of EBP‐Lips@si‐*Pear1* and Lips@si‐*Pear1* in the treatment of ALI, micro‐CT imaging was performed, and the results revealed that both EBP‐Lips@si‐*Pear1* and Lips@si‐*Pear1* significantly alleviated lung injury in septic mice, but the improvement was more pronounced in the EBP‐Lips@si‐*Pear1* group (Figure 7I). The results of the EBD extravasation assay and histopathological assessment were consistent with the above data (Figure 7J,K). As expected, the 72 h survival rate increased by 6.67% in the Lips@si‐*Pear1* group and more substantially by 33.33% in the EBP‐Lips@si‐*Pear1* group compared with that in the CLP group (Figure 7L). TEM analysis and determination of the protein expression of the cell junction‐related indicators ZO‐1, VE‐cadherin, Occludin, and Claudin‐1 revealed similar results (Figure 7M,N). Furthermore, compared with the CLP group, the levels of glycolysis‐related genes, H3K18la, and lactate in the lung tissue of mice in the EBP‐Lips@si‐*Pear1* group were significantly decreased (Figure S7L–N).

## 3 Discussion

**FIGURE 8:**
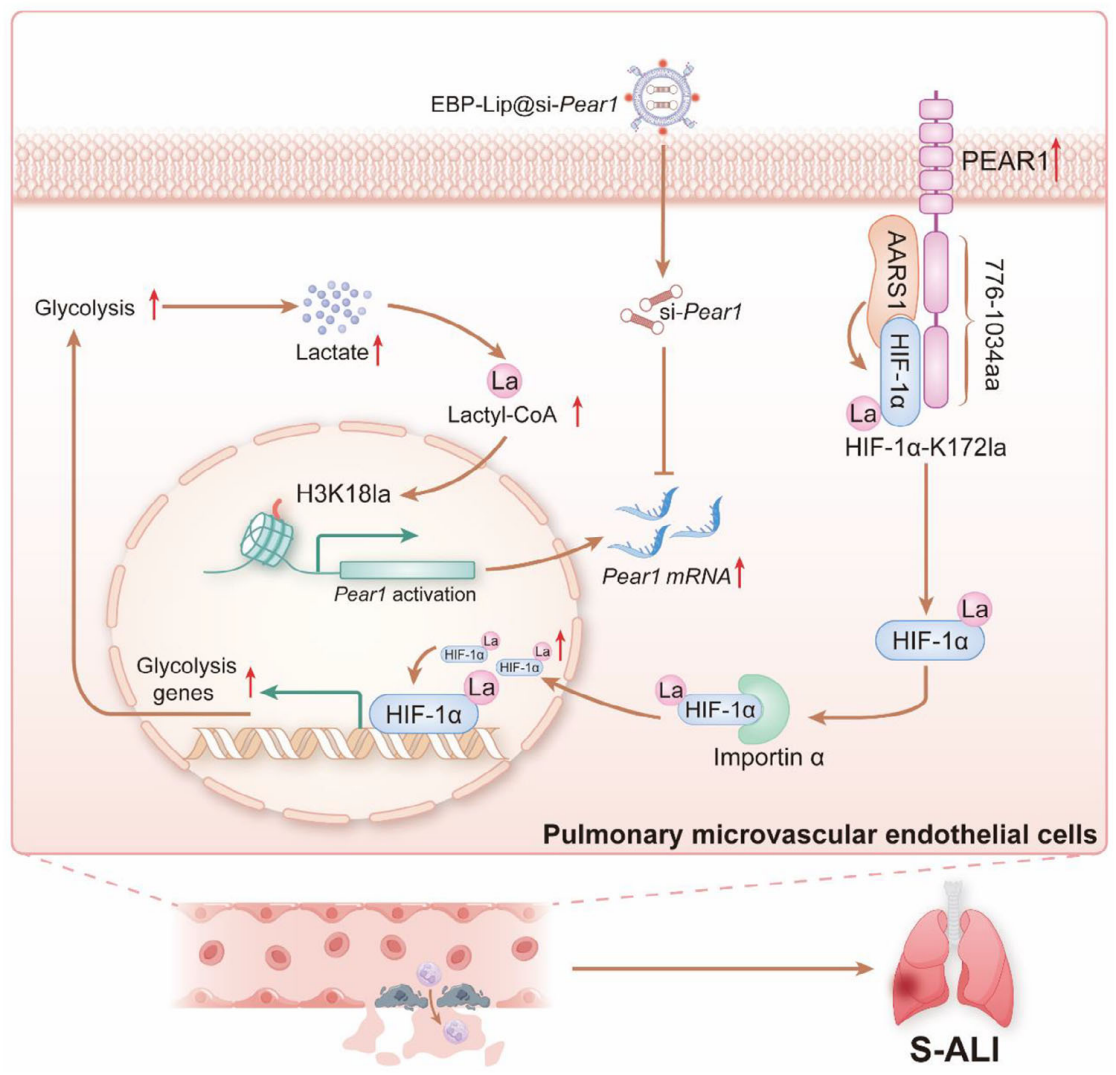
The central mechanism is that upregulated PEAR1 promotes AARS1‐mediated HIF‐1α lactylation at K172, enhances HIF‐1α interaction with importin α and nuclear translocation, and subsequently drives glycolysis and lactate production. The accumulated lactate further promotes H3K18 lactylation at the *Pear1* promoter, thereby enhancing *Pear1* transcription and establishing a positive feedback loop.

Although considerable advances have been made in elucidating the pathophysiology and therapy of S‐ALI, it remains a frequent and severe complication in critically ill patients and often contributes to unfavorable clinical outcomes [25, 40, 42]. S‐ALI arises from a network of interconnected pathophysiological processes in which immune dysregulation, vascular dysfunction, and metabolic adaptations collectively drive disease progression [43]. Nevertheless, how epigenetic mechanisms contribute to this complex interplay remains unclear. Our study revealed a PEAR1/HIF‐1α/glycolysis/lactate/H3K18la positive feedback loop in PMVECs that drives the development of S‐ALI. Specifically, during S‐ALI, elevated PEAR1 levels lead to increased vascular permeability. *Pear1* knockout alleviates lung injury and improves vascular permeability in mice. Mechanistically, PEAR1 mediates the binding of HIF‐1α to AARS1, leading to the lactylation of HIF‐1α, the primary lactylation site of which is K172. K172 lactylation of HIF‐1α promotes its interaction with importin α, thereby promoting HIF‐1α nuclear localization. Significantly, enhanced nuclear import of HIF‐1α stimulates glycolysis, and the lactate produced thereby increases H3K18 lactylation. This leads to H3K18la enrichment at the *Pear1* promoter, creating a positive feedback cycle that augments both glycolysis and pulmonary vascular permeability. In vivo, both the knockout of *Pear1* and the targeted delivery of si‐*Pear1* to inflammatory vascular endothelial cells mediated by E‐selectin‐binding peptide‐modified liposomes reduced pulmonary vascular permeability, alleviated ALI and significantly increased the survival rate of septic mice. A schematic summary of these mechanisms is shown in Figure 8.

The proliferation, migration and connections of PMVECs are key to the formation of pulmonary microvessels, which are involved in the regulation of permeability and are closely related to the occurrence and development of ARDS [44, 45, 46]. Lung imaging and histopathological analysis results revealed that 18 h after CLP, lung injury in mice was severe. These effects were accompanied by increased concentrations of inflammatory mediators in the BALF and serum lactate, as well as weakened cell junctions. In addition, in vitro experiments revealed that MPMVECs treated with 10 µg/mL LPS for 12 h exhibited a significant increase in cell permeability. PEAR1 is a transmembrane protein expressed in endothelial cells. To date, the role of PEAR1 signaling in the vascular endothelium has not been elucidated [8]. Our proteomic and scRNA‐seq studies in a CLP‐induced mouse lung injury model revealed marked increases in the levels of the PEAR1 protein and *Pear1* mRNA, most notably among PMVECs. Compared with those in HCs, the levels of circulating PEAR1 in patients with S‐ARDS were markedly elevated, and this increase was positively correlated with clinical prognostic indicators. Taken together, these data establish the pathogenic significance of PEAR1 in the development of S‐ALI. Furthermore, we used *Pear1* knockout mice and in vitro experiments to study the potential effect of PEAR1 on vascular permeability. *Pear1* knockout improved the survival of septic mice by enhancing cell junctions, reducing pulmonary vascular permeability, and mitigating lung tissue damage. These findings laid the foundation for our preliminary exploration of the role of PEAR1 in S‐ALI and PMVECs.

To elucidate the role of PEAR1 and its underlying mechanism in S‐ALI, we silenced *Pear1* in LPS‐stimulated MPMVECs for RNA‐seq and targeted energy metabolomics analyses. The results revealed significant alterations in glycolysis, and the above sequencing results were confirmed by WB, RT‐qPCR and cellular metabolism assays. These data suggest that LPS triggers PEAR1‐mediated metabolic reprogramming in PMVECs, which is characterized by enhanced glycolysis.

HIF‐1α serves as a central mediator that orchestrates glycolytic reprogramming and inflammatory responses, thereby linking metabolic dysfunction to the progression of lung diseases [47, 48]. Kuang et al. reported that inhibition of HIF‐1α/LDHA‐mediated immune metabolic disorders can markedly ameliorate endothelial permeability and ALI in sepsis [49]. This study revealed that *Pear1* knockdown did not affect the expression of HIF‐1α, either at the protein level or at the transcriptional level, but did affect the subcellular localization of HIF‐1α. Specifically, PEAR1 affects the entry of HIF‐1α into the nucleus by affecting the binding of HIF‐1α to importin α. While HIF‐1α activity is known to be modulated by PTMs, including hydroxylation, acetylation, and ubiquitination [50, 51], we revealed that HIF‐1α can promote its nuclear translocation through lactylation‐mediated regulation. These findings are consistent with our previous reports that KAT2B‐mediated EGR1 lactylation promotes its nuclear translocation and thereby accelerates endothelial glycocalyx degradation in septic mice [25]. It has also been reported that the lactylation of Snail1 by CBP/P300 facilitates endothelial‐to‐mesenchymal transition after myocardial infarction by promoting its nuclear translocation [52]. Our current findings further confirm that lactylation is a recurrent regulatory mechanism that promotes the nuclear translocation of transcription factors. In this study, *Pear1* overexpression specifically enhanced the association between K172la‐modified HIF‐1α and importin α. In contrast, the HIF‐1α K172R mutant inhibited this interaction and reduced nuclear translocation, confirming the essential role of K172 lactylation in this process. In summary, our results show that PEAR1 mediates the binding of HIF‐1α to importin α, enhancing HIF‐1α nuclear translocation and driving glycolysis and lactate production, which is an important mechanism through which PEAR1 regulates vascular permeability in the context of sepsis.

Despite growing reports of nonhistone protein lactylation (e.g., the lactylation of Ezrin, HMGB1, Snail1, YY1, and NSUN2), a comprehensive understanding of its regulatory dynamics and functional consequences remains largely elusive [52, 53, 54, 55, 56]. In contrast to those that catalyze histone lactylation, the enzymes that catalyze nonhistone lactylation are poorly characterized. Recently, AARS1 and AARS2 were identified as lactate sensors that mediate lactate transfer independent of lactyl‐CoA, acting as novel lactyltransferases [38, 39]. AARS1 participates in p53 lactylation, disrupting its liquid–liquid phase separation and DNA‐binding activity, a mechanism that contributes to tumorigenesis [39]. In both cellular and animal models, AARS2 binds to cyclic GMP‐AMP synthase (cGAS), mediating its lactylation and subsequent inactivation [38]. In this study, we showed that AARS1 can also act as a lactyltransferase and mediate HIF‐1α lactylation. These findings reveal a previously unrecognized role for AARS1 in regulating the pathogenesis of sepsis. Notably, the precise molecular mechanisms underlying AARS1 regulation and its functional connection to downstream glycolytic pathways warrant further investigation. AARS1 may also contribute to S‐ALI by catalyzing the lactylation of other proteins. Additionally, how AARS1 upregulates global lactylation in specific environments represents a promising direction for future exploration. From a clinical perspective, one limitation of this study is that septic patients without ARDS were not included as a disease control group; therefore, we cannot determine whether the increased serum PEAR1 level is specific to sepsis‐associated ARDS or reflects a general response to sepsis itself. Finally, siRNA‐based delivery knocks down total PEAR1 expression and thus cannot specifically disrupt the interaction sites between PEAR1 and AARS1 or HIF‐1α. Future studies will require network pharmacology‐based screening to identify site‐specific inhibitors, or the generation of transgenic mouse models expressing truncated or site‐mutated PEAR1 for further investigation.

In summary, our study identifies K172 as a previously unreported lactylation site on HIF‐1α and demonstrates that this modification promotes HIF‐1α nuclear translocation and glycolytic reprogramming during S‐ALI. We demonstrate that PEAR1 facilitates the AARS1‐mediated lactylation of HIF‐1α, which drives glycolysis. This metabolic shift, in turn, promotes *Pear1*/*PEAR1* transcription via H3K18 lactylation, thereby establishing a positive feedback loop. Collectively, these data provide mechanistic insight into the regulation of vascular permeability and establish the pathophysiological relevance of lactylation in driving S‐ALI progression.

## 4 Materials and Methods

### 4.1 Patients and Clinical Blood Samples

In accordance with the study protocol, whole blood samples from patients with sepsis‐associated acute respiratory distress syndrome (S‐ARDS) were collected within the first 12 h after admission to the Department of Respiratory and Critical Care Medicine at Anhui Chest Hospital between September 2023 and March 2025. Sepsis was diagnosed using the Sepsis‐3.0 criteria [2], and acute respiratory distress syndrome (ARDS) was diagnosed in accordance with the latest global definition [57]. The exclusion criteria included the following: 1) incomplete clinical information; 2) age < 18 years; 3) a diagnosis of malignant tumors or active tuberculosis; 4) severe hepatic or renal dysfunction, or coagulation disorders; 5) use of glucocorticoids or immunosuppressive agents; and 6) blood samples of inadequate quality or quantity. Blood samples from healthy controls (HCs) were obtained from the Medical Examination Center of Anhui Chest Hospital. A total of 83 patients with S‐ARDS and 24 HCs were ultimately enrolled in this study, which was conducted in accordance with the Declaration of Helsinki with approval from the Ethics Committee of Anhui Chest Hospital (Approval No. KJ2023‐40).

### 4.2 Animals

WT C57BL/6 mice were obtained from the Institute of Health and Medicine, Hefei Comprehensive National Science Center (Hefei, China). *Pear1*‐knockout (*Pear1* ^−^/^−^) mice were generated by Cyagen Biosciences Inc. (Suzhou, China) using CRISPR/Cas9‐mediated gene editing with the following gRNA target sequences:

gRNA‐A1: CTCGGTCCAGAACTTTCCGATGG,

gRNA‐A2: ACAGAGAAGTGAACGGCAATGGG,

gRNA‐B1: GTTAGGCTAGGGGGTGTTCAGGG,

gRNA‐B2: CCAGGATGTATGACTAGGCAAGG.

All mice were housed and bred within the specific pathogen‐free (SPF) animal facility of the Hefei Comprehensive National Science Center. Genotyping was performed by polymerase chain reaction (PCR) amplification of genomic DNA extracted from tail biopsies. Age‐ and sex‐matched male mice between 8 and 12 weeks of age were used for all the experiments, which were conducted in a blinded manner. All animal experiments were conducted in strict compliance with the Experimental Animal Management Ordinance of China and were reviewed and approved by the Institutional Animal Ethics Committee of the Hefei Comprehensive National Science Center (IHM‐AP‐2024‐004).

### 4.3 Cecal Ligation and Puncture (CLP) Induced Polymicrobial Sepsis Model

The mice were randomly allocated to experimental groups, and the investigators were blinded to group identity throughout the study to minimize bias. Polymicrobial sepsis in mice was induced by CLP as previously described [13]. Before surgery, the mice were anesthetized with 4% isoflurane, after which the abdominal area was shaved and disinfected. Following a 1 cm midline laparotomy, the cecum was carefully exteriorized and ligated between the third and fourth vascular arches with a 4‐0 silk suture. The ligated cecum was punctured once with a 20‐gauge needle to allow extrusion of a small amount of fecal content. Sham‐operated control animals underwent the same surgical procedure, excluding cecal ligation and puncture. Postoperatively, the mice received a single subcutaneous dose of resuscitation fluid. Blood and lung tissue samples were harvested at predetermined time points according to the experimental protocol.

### 4.4 Single‐Cell RNA Sequencing (scRNA‐seq)

Single‐cell suspensions were prepared from the lung tissues of CLP and sham‐operated mice, after which scRNA‐seq and bioinformatics analyses were conducted by Chi‐Bio Technology (Guangzhou, China; http://www.chi‐biotech.com). Single‐cell suspensions were generated from lung tissues using a standard protocol. The cell viability was confirmed to be >90% by Trypan blue exclusion, and viable cells were resuspended at a concentration of 1.0–1.2 × 10^6^ cells/mL for downstream processing. Following single‐cell capture on the DNBelab C4 platform, cDNA amplification and library preparation were performed according to the manufacturer's protocol (BGI‐Shenzhen, China), and the resulting libraries were subjected to sequencing.

Raw FASTQ files were subjected to quality control (SOAPnuke) and aligned to the GRCh38/mm10 genome using the DRAGEN Bio‐IT Platform to generate a cell‐by‐gene UMI count matrix. Downstream analysis was performed in R/Seurat. Cells were filtered on the basis of unique gene counts (200–6,000) and the mitochondrial ratio (<20%). After the multiplexed cells were removed and low‐quality cells were filtered, a total of 20,584 single cells were obtained, including 10,139 cells from CLP mouse lung tissue and 10,445 cells from SHAM mouse lung tissue. The data were log‐normalized, and the top 2,000 highly variable genes (HVGs) were subjected to principal component analysis (PCA). Following Harmony integration of the top 30 PCs to correct for batch effects, the cells were clustered (Louvain) and visualized (UMAP), yielding annotated and quantified populations across conditions.

### 4.5 PMVECs Culture

Primary MPMVECs, which were originally isolated from C57BL/6 mouse lung tissue, were purchased from Procell Biotechnology Co., Ltd. (Wuhan, China) and cultured in complete endothelial cell medium supplemented with 5% FBS, 1% penicillin‐streptomycin, and 1% endothelial cell growth supplement at 37°C with 5% CO_2_. For isolation, lungs were perfused with pre‐cooled DMEM containing 1% FBS, excised, minced into 1 mm^3^ fragments, and placed in culture flasks to allow cell attachment. After 2 h, medium was added, and tissues were cultured for 96 h. Thereafter, the medium was replaced every 48 h. The migrating cells were collected, and tissue fragments were removed; the cells were subsequently expanded and passaged for experiments.

HPMECs were purchased from EnkiLife Biotechnology Co., Ltd. (Wuhan, China) and were originally isolated from human lung tissue using a dissociation enzyme mixture. Cells were cultured in EnkiLife complete primary endothelial cell medium at 37°C with 5% CO_2_.

To simulate an inflammatory environment in vitro, the cells were treated with 10 µg/mL E. coli‐derived LPS for 12 h.

### 4.6 Cell Viability Assay

Cell viability was determined by a CCK‐8 assay according to the manufacturer's instructions. MPMVECs were plated in 96‐well plates and treated under the indicated conditions. After treatment, 10 µL of CCK‐8 reagent was added to each well, and the plates were incubated for 2 h at 37°C in the dark. The optical density at 450 nm was subsequently measured using a microplate reader (PerkinElmer, USA).

### 4.7 Micro‐Computed Tomography (Micro‐CT) Analysis

Longitudinal monitoring of acute lung injury progression was performed in live mice using the Hiscan XM Micro‐CT system. Thoracic scans were acquired at the indicated timepoints for quantitative assessment of injury severity. Images were acquired at a 50 µm resolution using a micro‐CT system set to 60 kV/134 µA, employing a 0.5° rotation step over a full 360° scan with 50 ms of exposure per projection. Image processing and volumetric rendering were conducted using Hiscan Analyzer Software (version 3.0; Hiscan Information Technology Co., Ltd., China).

### 4.8 Hematoxylin and Eosin (H&E) Staining and Evaluation of Lung Injury Severity

Lung tissue samples were fixed in 4% paraformaldehyde (PFA; ServiceBio, G1101) and subsequently embedded in paraffin. The formalin‐fixed, paraffin‐embedded (FFPE) blocks were cut into 4 µm thick sections using a microtome. The tissue sections were then stained with H&E for histological examination. Histopathological lung injury was evaluated using a semiquantitative scoring system based on previously described methods, with minor modifications [58]. Four parameters were assessed on a scale of 0–4 according to the extent of involvement (0, no injury; 1, ≤25%; 2, ≤50%; 3, ≤75%; 4, diffuse involvement): alveolar edema, hemorrhage, neutrophil infiltration, and hyaline membrane formation.

### 4.9 Bronchoalveolar Lavage Fluid (BALF) Cell Counting and Classification

Bronchoalveolar lavage was performed via three sequential instillations of 0.5 mL of sterile saline per mouse and pooled in a single collection tube. A 50 µL aliquot of BALF was analyzed using an XT‐2000i automated hematology analyzer (Sysmex, Japan) for total cell counting. Additionally, cell counting was performed on cytospin preparations stained with modified Giemsa stain (Beyotime Biotechnology, China).

### 4.10 In Vivo Vascular Permeability Assay

#### 4.10.1 Vascular Permeability was Evaluated Using Two Complementary Methods

To assess pulmonary capillary permeability, Evans blue dye (EBD; 50 mg/kg) was injected intravenously 1 h before sacrifice. Vascular permeability was determined by measuring EBD extravasation into the lung tissue. To remove intravascular dye, systemic perfusion was performed with 0.9% saline through the left ventricle until the effluent became clear. Following harvest, the lungs were first weighed and then homogenized in 200 µL of formamide. The homogenate was then incubated at 56°C for 24 h to allow for complete extraction of the EBD. After centrifugation (12,000 × g, 10 min), the optical density of the supernatant was measured at 620 nm, and this value served as an indicator of vascular leakage.

#### 4.10.2 Pulmonary Capillary Permeability Index Determination

The protein concentrations in the BALF and plasma were quantified with a BCA assay kit (Beyotime Biotechnology, Cat# P0012). The lung permeability index was subsequently determined from the BALF/plasma protein ratio to assess vascular leakage.

### 4.11 Transmission Electron Microscopy (TEM)

Eighteen hours after surgery, the CLP and sham‐operated mice were placed under deep anesthesia and subjected to transcardial perfusion with fixative solution. The lung tissue was collected and cut into 1 mm^3^ pieces. The lung tissue pieces were fixed with precooled 2.5% glutaraldehyde, rinsed with phosphate buffer and fixed with 1% osmic acid. After being rinsed again, the samples were subjected to staining, dehydration in graded ethanol, and embedding in Epon 812. Ultrathin sections were sequentially stained with uranyl acetate for 15 min and lead citrate for 2 min prior to imaging on a Thermo Scientific Talos L120C G2 transmission electron microscope.

### 4.12 In Vitro Permeability Assay

TEER was measured to evaluate endothelial barrier permeability using the Millipore Millicell ERS‐2 ECIS system. Cells were cultured to confluence on Transwell inserts, and baseline measurements were taken 1 h prior to experimental treatment. Subsequent measurements were taken at 12 h post‐LPS exposure to assess changes in MPMVEC permeability.

### 4.13 Enzyme‐Linked Immunosorbent Assay (ELISA)

We quantified serum PEAR1 concentrations in S‐ARDS patients and HCs with a commercially available ELISA kit (Abcam, ab313922) according to the manufacturer's protocol. Additionally, lactate concentrations in mouse serum, lung tissue and cultured cells were determined using a commercial Lactate Assay Kit (Elabscience, E‐BC‐K044‐S). The concentrations of IL‐6, TNF‐α, and IL‐1β in both the cell culture supernatants and BALF were measured using commercially available ELISA kits (Elabscience, E‐EL‐M0044; Elabscience, E‐EL‐M3063; Elabscience, E‐EL‐M0037, respectively).

### 4.14 Immunofluorescence (IF) Staining

Lung tissue samples from mice were fixed in 4% paraformaldehyde (PFA), paraffin‐embedded, and sectioned at a thickness of 4 µm. Following deparaffinization, antigen retrieval was performed by heating the sections in Tris‐EDTA buffer (pH 8.0; ServiceBio, G1206) for 2.5 min using a pressure cooker. MPMVECs grown on coverslips were washed three times with PBS and then fixed with 4% PFA. Both lung tissue and cells underwent permeabilization and blocking steps. Thereafter, they were sequentially incubated with the primary antibody, secondary antibody, and DAPI. Finally, the samples were mounted and image acquisition and analysis were performed with an LSM‐980 laser scanning confocal microscope (Zeiss, Germany) and ImageJ software, respectively. Additional details were provided in our previous article [25].

### 4.15 Proximity Ligation Assay (PLA)

MPMVECs grown on coverslips were fixed in 4% PFA (37°C, 15 min) and then permeabilized with 0.2% Triton X‐100 in PBS (room temperature, 15 min). The NaveniFlex Cell MR Atto647N assay was subsequently performed following the manufacturer's instructions. Briefly, cells were coincubated with mouse anti‐HIF‐1α (Abcam, ab1, 1:100) and rabbit anti‐importin‐α (Abcam, ab307438, 1:50) primary antibodies. After signal development, the nuclei were stained with DAPI. Specific spots representing molecular interactions were visualized with a Zeiss LSM980 confocal system.

### 4.16 Nuclear‐Cytoplasmic Protein Separation

MPMVECs were washed with PBS, harvested by scraping, and pelleted by centrifugation (300 × g, 10 min, 4°C). Cellular proteins were fractionated into nuclear and cytoplasmic components using a commercial extraction kit (Beyotime Biotechnology, Cat# P0027). Protein samples were then analyzed by immunoblotting with specific primary antibodies.

### 4.17 Immunoprecipitation (IP)

We performed IP with the Pierce Classic Magnetic IP/Co‐IP Kit (Thermo Fisher, #88804). The cell lysates were subjected to overnight incubation with primary antibody at 4°C, followed by a 1‐hour incubation with protein A/G magnetic beads at room temperature with gentle agitation to capture the immune complexes. Finally, the bound proteins were eluted for downstream applications, including western blot (WB) analysis and mass spectrometry.

### 4.18 Liquid Chromatography–Tandem Mass Spectrometry (LC–MS/MS)

LC–MS/MS was used to identify HIF‐1α lactylation sites and interacting proteins in MPMVECs. The samples were processed on ice using a high‐intensity ultrasonic processor (Scientz). The lysis buffer consisted of 8 M urea, 3 µM trichostatin A (TSA), 50 mM nicotinamide (NAM), 2 mM EDTA, and 1% protease inhibitor cocktail. The lysate was centrifuged (12,000 × g, 10 min, 4°C), and the resulting supernatant was quantified via a BCA assay. For proteomic analysis, proteins were reduced with 5 mM DTT (37°C, 60 min) and alkylated with 10 mM IAA (room temperature, 45 min in the dark). Details of these steps were provided in our previous article [25].

### 4.19 CUT&Tag

Library preparation for CUT&Tag sequencing was performed using a NovoNGS CUT&Tag 4.0 High‐Sensitivity Kit (NovoProtein, N259‐YH01) following the manufacturer's instructions. Following immobilization on concanavalin A‐coated magnetic beads, permeabilization, and resuspension in antibody buffer, 50,000 MPMVECs were sequentially incubated with primary antibodies against H3K18la (PTM‐1427RM, PTM Bio) and H3K18ac (PTM‐114RM, PTM Bio) and their respective secondary antibodies. Details of these steps were provided in our previous article [25].

### 4.20 RNA‐seq and Data Analysis

Total RNA was extracted from MPMVECs using TRIzol reagent following the manufacturer's protocol. Following extraction, ribosomal RNA (rRNA) was depleted, and the remaining RNA was processed for cDNA library preparation. The libraries were constructed and sequenced on the DNBSEQ platform (MGI Tech). Bioinformatic analysis was performed to identify differentially expressed genes (DEGs) using DEG‐seq with significance thresholds of |log2FC| > 1 and a *p* value < 0.05. Sequencing and computational analyses were conducted by Huada Gene Company (Wuhan, China).

### 4.21 Western Blot (WB) Analysis

Protein samples extracted from cells and tissues were denatured, separated by SDS‐PAGE, and transferred onto PVDF membranes (Millipore, USA). Following blocking with 5% skim milk (1.5 h, room temperature), the membranes were incubated with primary antibodies overnight at 4°C. After they were washed, the membranes were incubated with HRP‐conjugated secondary antibodies for 1 h at room temperature. Signal detection was subsequently performed using a Tanon 5200 chemiluminescence imaging system (China), and quantification was carried out using ImageJ software. The antibody details were given in Table S2.

### 4.22 Reverse Transcription Quantitative Polymerase Chain Reaction (RT‐qPCR)

Total RNA was isolated (HiPure Total RNA Mini Kit; Magen, R4111), quantified (NanoDrop One), and reverse‐transcribed (HiScript IV RT SuperMix; Vazyme Biotech, R423) according to the manufacturers' instructions. qPCR was performed in triplicate on a Roche LightCycler 480 II system using ChamQ SYBR Master Mix (Vazyme Biotech, Q311), and target expression was normalized to that of GAPDH. The primer sequences used were listed in Table S3.

### 4.23 Molecular Docking

The protein structures of mouse HIF‐1α, AARS1, and PEAR1 were downloaded from the UniProt database (https://www.uniprot.org/). All protein structures were processed using PyMOL 2.3.0 software, including the removal of water molecules, ions, and other solvated molecules. The interactions between HIF‐1α and AARS1, between PEAR1 and HIF‐1α, and between PEAR1 and AARS1 were studied using ZDOCK. The molecular docking results were further characterized with PyMOL (v2.3.0) to delineate the specific interaction patterns.

### 4.24 Chromatin Immunoprecipitation (ChIP) Assay

ChIP experiments were performed using a ChIP assay kit (LABLEAD, CP0581). The specific procedure was as follows. After cross‐linking with 1% formaldehyde at 37°C for 10 min, approximately 1×10^6^ PMVECs were subjected to chromatin fragmentation by ultrasonication. The soluble chromatin lysate was incubated with a target‐specific primary antibody overnight at 4°C for immunoprecipitation, with normal IgG serving as a negative control for nonspecific binding. The samples were then gently shaken with protein A/G magnetic beads at 4°C for 60 min, followed by washing to elute the antibody‐bound protein–DNA complex. Finally, the immunoprecipitated DNA was analyzed via ChIP‐PCR or ChIP‐qPCR using specific primers targeting the promoter region (Table S5).

### 4.25 Protein Expression and Purification

His‐tagged HIF‐1α protein or His‐tagged AARS1 was expressed in *E. coli* BL21(DE3) cells transformed with pET28a‐HIF‐1α/AARS1. Bacterial cultures were grown overnight at 37°C before protein expression was induced using 1 mM IPTG at 18°C for 1 h. The cells were collected by centrifugation (1,000 × g) and lysed via sonication in His lysis buffer (25 mM Tris‐HCl (pH 8.0), 300 mM NaCl, 20 mM imidazole, 5% glycerol, and 1 mM TCEP). After centrifugation, the clarified supernatant was loaded onto a preequilibrated HisPur Ni‐NTA resin column (5 mL; Thermo Fisher, cat. 90099). The column was subsequently washed with imidazole‐containing lysis buffer (excluding glycerol and TCEP), and the bound protein was eluted using His elution buffer (25 mM Tris‐HCl, pH 8.0; 300 mM NaCl; 400 mM imidazole). Finally, the purified protein was buffer‐exchanged into PBS for downstream applications.

To express GST‐tagged AARS1, GST‐AARS1(Δ846‐939 aa), or GST‐PEAR1(776‐1034 aa), *E. coli* BL21(DE3) cells transformed with the corresponding pGEX‐4T‐1 plasmids were grown overnight at 37°C. Protein expression was then induced with 1 mM IPTG at 18°C for 1 h. Following centrifugation at 1,000 × g, the cells were lysed by sonication in GST lysis buffer. The lysate was clarified by centrifugation, and the supernatant was subjected to affinity purification on a preequilibrated Pierce glutathione column (5 mL). After being washed with GST lysis buffer (without glycerol or TCEP), the bound protein was eluted using GST elution buffer containing 40 mM reduced glutathione. Finally, the purified proteins were buffer‐exchanged into PBS.

To purify of eukaryotic His‐tagged AARS1, His‐tagged HIF‐1α, and the GST‐tagged intracellular domain of PEAR1, the corresponding plasmids were transfected into HEK293T cells for protein expression. The cell lysate was subjected to protein purification using a protocol identical to that for prokaryotic systems.

### 4.26 GST Pull‐Down Assay

The GST‐AARS1, GST‐AARS1(Δ846‐939 aa), His‐HIF‐1α, GST‐PEAR1(776‐1034 aa), and His‐AARS1 proteins were purified for the GST pull‐down assays. GST and GST‐fused bait proteins were immobilized onto glutathione (GSH)‐coupled magnetic beads using a Pierce GST Pull‐Down Kit (Thermo Fisher, cat: 21516) following the manufacturer's protocol. The immobilized proteins were then incubated with a His‐tagged protein to assess protein‐protein interactions. After being washed, the bound complexes were eluted and analyzed.

### 4.27 In Vitro Lactylation Assays

Following the methods of Li et al. [38], the lactylation reaction was performed in buffer containing 50 mM HEPES (pH 7.5), 25 mM KCl, 2.0 mM MgCl_2_, 2 mM L‐lactate, 50 nM AARS1, 2 mM ATP, and 0.1 µg/µL substrate (HIF‐1α or PEAR1(776‐1034 aa)). The mixture was incubated at 37°C for 1 h. The products were then separated by 10% SDS–PAGE and detected by WB analysis.

### 4.28 Plasmids and Transfection

The overexpression plasmids pcDNA3.1(+)‐3Flag‐*Pear1* and pcDNA3.1(+)‐3HA‐*Aars1* were constructed using the cDNA of *Pear1* (NC_000069.7) and *Aars1* (NC_000074.7) and transfected with the corresponding empty vector. By using full‐length *Pear1* cDNA or *Aars1* cDNA as a template, a series of truncated plasmids, including Flag‐*Pear1*‐(19‐754aa), Flag‐*Pear1*‐(776‐1034aa), and HA‐*Aars1*‐(Δ846‐939aa), were cloned and inserted into pcDNA3.1(+)‐3Flag or pcDNA3.1(+)‐3HA. Site‐directed mutagenesis was performed with specific primers to generate *Hif1a* mutants (K11R, K12R, and K172R), which were subsequently subcloned and inserted into a pcDNA3.1 vector to produce N‐terminal 6×His‐tagged fusion proteins. Transfection was carried out with Lipofectamine 3000 according to the manufacturer's instructions. The siRNAs were designed and synthesized by GenePharma Co., Ltd. (Shanghai, China). We transfected MPMVECs with CALNP RNAi transfection reagent (D‐Nano Therapeutics, DN001) in accordance with the recommended protocol. The target sequences of the siRNAs were as follows: mouse siRNA‐*Pear1*, #1 CCGUUGCUUUCCUGCUAAUTT, #2 AUUAGCAGGAAAGCAACGGTT; human si‐RNA‐*PEAR1*, #1 GCACGCUGCUCAUGUGAAATT, #2 UUUCACAUGAGCAGCGUGCTT, and mouse siRNA‐*Aars1*, #1 GGGACTATTTCAAGGAATT, #2 GGGAAGCCCTCTCGGGAAA.

### 4.29 Real‐Time Cell Metabolism Assay

The oxygen consumption rate (OCR) and the extracellular acidification rate (ECAR) were quantified using a Seahorse XFe96 Extracellular Flux Analyzer (Agilent Technologies) according to the manufacturer's protocols. Briefly, MPMVECs were plated in XF96 microplates (2,000 cells/well) and stimulated for 24 h with LPS or media as a control. Following the manufacturer's instructions, we simultaneously monitored the ECAR and OCR in real time. The OCR was measured at baseline and following defined mitochondrial perturbations: oligomycin (1.5 µM), FCCP (1.0 µM), and rotenone/antimycin A (0.5 µM each). The ECAR was quantified before and after glycolysis inhibition with 2‐DG. All measurements were normalized on the basis of the total protein concentration.

### 4.30 Cellular Energy Metabolism Analysis

Cell metabolites were analyzed by targeted energy metabolomics by Shanghai Personal Biotechnology Co., Ltd. Briefly, samples that had been stored at ‐80°C were thawed on ice, and the cells were evenly resuspended in 100 µl of ultrapure water. Afterward, 50 µl of the cell suspension was added to 200 µl of pure methanol extract and vortexed for 2 min at 2500 r/min; this step was repeated 3 times. The sample was then centrifuged at 12000 r/min and 4°C for 10 min, after which 200 µl of the supernatant was placed in a new centrifuge tube. After incubation at ‐20°C for 30 min followed by centrifugation at 4°C and 12000 r/min for 10 min, 180 µl of the supernatant was passed through a protein precipitation plate for subsequent analysis. Data collection was performed using an ultrahigh‐performance liquid chromatograph (ACQUITY H‐ClassD, Waters, USA) and a tandem mass spectrometer (QTRAP6500 +, SCIEX, USA). Quantitative analysis was carried out using the multiple reaction monitoring mode of the triple quadrupole mass spectrometer. Finally, the raw mass spectrometry data were processed using Analyst 1.6.3 software. The distribution of metabolites was analyzed by constructing a heatmap and volcano plot.

### 4.31 Nanomaterial Synthesis

E‐selectin‐binding peptide‐modified cationic liposomes/cationic liposomes loaded with small interfering RNA:

E‐selectin‐binding peptide was reacted with DSPE‐PEG‐NHS at pH 8, and the DSPE‐PEG‐peptide solution was obtained after dialysis. Then, DOTAP, DOPE, cholesterol, and DID with DSPE‐PEG‐NHS or DSPE‐PEG‐peptide were dissolved in 1 mL of anhydrous ethanol. *Pear1*‐targeted small interfering RNA (si‐*Pear1*) was dissolved in citrate buffer (50 mM citrate, pH 4), and the liposome solution was mixed with citric acid solution and incubated for 20 min. After sonication and extrusion through a liposome extruder (100 nm filter membrane), unloaded nucleic acid was removed by dialysis. Finally, the volume was adjusted to 3 mL by adding deionized water, and a specific amount of freeze‐drying protective agent was added before the sample was freeze dried.

### 4.32 Adeno‐Associated Virus (AAV) Construction and In Vivo Administration

To achieve endothelial‐specific knockdown of *Pear1* with minimal cytotoxicity, an adeno‐associated virus serotype 9 (AAV9) vector was constructed. The shRNA sequence targeting *Pear1* was embedded into a miR30 backbone under the control of the endothelial‐specific Icam‐2 promoter (AAV9‐*Icam‐2*‐miR30‐sh‐*Pear1*‐GFP, termed AAV‐sh‐*Pear1*). An AAV9 vector carrying a scramble shRNA sequence (AAV9‐sh‐NC) was used as a negative control to exclude non‐specific effects of the viral transduction and shRNA expression system. All the vectors were packaged and purified by GenePharma (Suzhou, China). Male mice received a single tail‐vein injection of AAV9 at a dose of 5 × 10^1^ ^1^ vg per mouse. The experimental procedures were performed 4 weeks after viral delivery to ensure stable and efficient gene silencing.

### 4.33 Statistical Analysis

The data were presented as the mean ± standard deviation (SD). Differences between two groups were compared using an unpaired Student's t test. Comparisons among three or more groups were performed by one‐way ANOVA followed by the Tukey–Kramer post hoc test. Survival differences were determined using the Kaplan–Meier method and the log‐rank test. All analyses were performed using the Graph Pad Prism 8.0 statistical software package, and *p* < 0.05 was considered to indicate statistical significance. ^*^ *p* < 0.05, ^**^ *p* <0.01, ^***^ *p* < 0.001, and ^****^ *p* < 0.0001; ns: not significant.

For clinical data, normality was first assessed using the D'Agostino test. Normally distributed continuous variable data were presented as the mean ± SD and were compared using one‐way ANOVA. Nonnormally distributed data were presented as medians (interquartile ranges, IQRs) and were analyzed with the Kruskal–Wallis test. Categorical variables were expressed as frequencies (percentages) and were compared using the chi‐square test or Fisher's exact test, as appropriate. Correlations were assessed using Pearson's test for parametric data and Spearman's method for nonparametric data.

## Author Contributions

**S.L**.: Writing – review & editing, writing – original draft. **Z.L**., **P.F**., **Q.P**.: Visualization, validation. **X.W**., **B.W**. and **M.L**.: Supervision, software. **J.P**. and **H.K**.: Resources. **H.C**.: Project administration, methodology. **J.L**., **J.K**.: Investigation, formal analysis, data curation. **D.W**.: Data curation. **D.X**.: Data curation, conceptualization. **M.X**. and **Q.Y**.: Funding acquisition, formal analysis. All authors approved the final version of the manuscript.

## Conflicts of Interest

The authors declare no conflicts of interest.

## Supporting information




**Supporting File**: advs76592‐sup‐0001‐SuppMat.docx.

## Data Availability

The data that support the findings of this study are available from the corresponding author upon reasonable request.
