# Supplementary material for: PEAR1 Promotes Glucose Metabolism Reprogramming in Sepsis‐Associated Acute Lung Injury via AARS1‐Mediated HIF‐1α Lactylation
Source: Adv Sci (Weinh). 2026 Jul 14;13(56):e76592. doi: 10.1002/advs.76592 (PMC13366482; doi:10.1002/advs.76592)
Supplement: Supplementary file 1 [file ADVS-13-e76592-s001.docx]

**Table S1 The characteristics of sepsis-related ARDS patients with different severity.**

| Variables | Total (n = 83) | Mild (n = 18) | Moderate (n = 33) | Severe (n = 32) | *P* value |  |
| --- | --- | --- | --- | --- | --- | --- |
| Gender, n (%) |  |  |  |  | 0.445 |  |
| Male | 60 (72.3%) | 15 (18.07%) | 22 (26.51%) | 23 (27.71%) |  |  |
| Female | 23 (27.7%) | 3 (3.61%) | 11 (13.25%) | 9 (10.84%) |  |  |
| Age (years) | 76.00 (67.50, 81.00) | 80.50 (68.00, 82.75) | 74.00 (66.00, 80.00) | 76.00 (66.50, 80.25) | 0.319 |  |
| Hypertension, n (%) |  |  |  |  | 0.469 |  |
| No | 53 (63.9%) | 11 (13.25%) | 19 (22.89%) | 23 (27.71%) |  |  |
| Yes | 30 (36.1%) | 7 (8.43%) | 14 (16.87%) | 9 (10.84%) |  |  |
| Diabetes mellitus, n (%) |  |  |  |  | 0.886 |  |
| No | 67 (80.7%) | 15 (18.07%) | 27 (32.53%) | 25 (30.12%) |  |  |
| Yes | 16 (19.3%) | 3 (3.61%) | 6 (7.23%) | 7 (8.43%) |  |  |
| COPD, n (%) |  |  |  |  | 0.260 |  |
| No | 68 (81.9%) | 14 (16.87%) | 25 (30.12%) | 29 (34.94%) |  |  |
| Yes | 15 (18.1%) | 4 (4.82%) | 8 (9.64%) | 3 (3.61%) |  |  |
| ACS, n (%) |  |  |  |  | 0.027* |  |
| No | 69 (83.1%) | 14 (16.87%) | 24 (28.92%) | 31 (37.35%) |  |  |
| Yes | 14 (16.9%) | 4 (4.82%) | 9 (10.84%) | 1 (1.20%) |  |  |
| Cerebrovascular, n (%) |  |  |  |  | 0.063 |  |
| No | 70 (84.3%) | 12 (14.46%) | 29 (34.94%) | 29 (34.94%) |  |  |
| Yes | 13 (15.7%) | 6 (7.23%) | 4 (4.82%) | 3 (3.61%) |  |  |
| SOFA score | 5.00 (4.00, 8.00) | 4.50 (4.00, 5.00) | 5.00 (4.00, 7.00) | 7.50 (4.00, 9.00) | 0.017* |  |
| APACHE II score | 16.67 ± 7.72 | 12.67 ± 4.31 | 16.48 ± 7.85 | 19.12 ± 8.26 | 0.016* |  |
| Septic shock, n (%) |  |  |  |  | 0.789 |  |
| No | 60 (72.3%) | 14 (16.87%) | 24 (28.92%) | 22 (26.51%) |  |  |
| Yes | 23 (27.7%) | 4 (4.82%) | 9 (10.84%) | 10 (12.05%) |  |  |
| Mortality_28day, n (%) |  |  |  |  | 0.053 |  |
| No | 42 (50.6%) | 12 (14.46%) | 19 (22.89%) | 11 (13.25%) |  |  |
| Yes | 41 (49.4%) | 6 (7.23%) | 14 (16.87%) | 21 (25.30%) |  |  |
| ICU stay duration (days) | 11.00 (6.00, 17.50) | 10.00 (7.00, 15.50) | 15.00 (9.00, 23.00) | 6.50 (5.00, 11.25) | 0.004* |  |
| pH | 7.45 (7.36, 7.48) | 7.46 (7.42, 7.52) | 7.45 (7.38, 7.48) | 7.43 (7.32, 7.47) | 0.357 |  |
| PaCO_2_ (mmHg) | 38.70 (31.25, 54.15) | 38.35 (34.02, 49.58) | 42.10 (33.70, 61.90) | 37.65 (27.75, 50.95) | 0.432 |  |
| PaO_2_/FiO_2_ (mmHg) | 126.00 (89.00, 188.50) | 234.50 (216.75, 266.98) | 146.90 (124.40, 168.00) | 83.50 (64.80, 92.25) | <0.001* |  |
| Lactate (mmol/L) | 2.02 (1.20, 4.35) | 1.20 (0.83, 1.87) | 2.19 (1.13, 4.68) | 3.08 (1.58, 6.60) | <0.001* |  |
| WBC count (10^9^/L) | 10.33 (8.32, 13.25) | 10.15 (8.43, 14.04) | 9.78 (7.32, 11.91) | 10.84 (8.75, 14.80) | 0.391 |  |
| Neutrophil count (10^9^/L) | 8.83 (7.03, 11.46) | 8.07 (7.28, 10.99) | 8.63 (6.25, 10.56) | 9.93 (7.09, 13.70) | 0.440 |  |
| Lymphocyte count (10^9^/L) | 0.62 (0.36, 0.95) | 0.78 (0.45, 0.98) | 0.59 (0.40, 0.95) | 0.60 (0.30, 0.95) | 0.500 |  |
| Hemoglobin (g/L) | 107.27 ± 24.89 | 100.00 ± 27.30 | 110.82 ± 24.10 | 107.69 ± 24.22 | 0.334 |  |
| Platelet (10^9^/L) | 197.33 ± 114.77 | 195.94 ± 111.74 | 193.36 ± 122.14 | 202.19 ± 112.05 | 0.953 |  |
| CRP (mg/L) | 83.63 (35.52, 149.16) | 81.30 (36.87, 127.15) | 85.58 (24.20, 144.45) | 71.26 (40.17, 165.69) | 0.837 |  |
| PCT (ng/mL) | 0.52 (0.14, 2.24) | 0.41 (0.16, 1.21) | 0.38 (0.09, 1.82) | 1.35 (0.26, 4.31) | 0.068 |  |
| Fibrinogen (g/L) | 4.22 (2.54, 5.55) | 3.69 (2.22, 5.67) | 4.22 (2.83, 5.40) | 4.47 (2.56, 5.54) | 0.834 |  |
| D-dimer (mg/L) | 2.25 (0.83, 4.29) | 2.14 (0.99, 3.87) | 1.21 (0.58, 4.22) | 3.54 (1.39, 4.66) | 0.286 |  |
| Albumin (g/L) | 29.35 ± 6.51 | 30.05 ± 4.07 | 31.63 ± 6.33 | 26.61 ± 6.93 | 0.006* |  |
| ALT (U/L) | 25.00 (17.00, 55.00) | 24.00 (15.75, 48.75) | 24.00 (16.00, 57.00) | 27.00 (19.50, 53.00) | 0.696 |  |
| AST (U/L) | 39.00 (28.50, 64.50) | 46.00 (32.25, 61.25) | 33.00 (24.00, 59.00) | 41.50 (30.75, 71.50) | 0.275 |  |
| Bilirubin (mg/dL) | 12.20 (8.95, 17.80) | 12.00 (9.27, 16.25) | 12.20 (9.20, 17.60) | 11.80 (8.38, 19.03) | 0.934 |  |
| BUN (mmol/L) | 9.00 (6.90, 12.95) | 10.30 (7.80, 20.12) | 12.20 (9.20, 17.60) | 11.80 (8.38, 19.03) | 0.321 |  |
| Creatinine (μmol/L) | 66.90 (48.45, 106.70) | 71.30 (51.47, 113.12) | 67.50 (45.60, 91.50) | 64.35 (51.77, 107.60) | 0.671 |  |
| Glucose (mmol/L) | 7.79 (6.60, 10.69) | 7.10 (6.43, 8.19) | 7.68 (6.69, 10.59) | 9.00 (6.85, 12.31) | 0.053 |  |
| PEAR1 (ng/mL) | 42.03 ± 10.43 | 32.12 ± 5.87 | 38.57 ± 5.55 | 51.18 ± 8.98 | <0.001* |  |

COPD: Chronic Obstructive Pulmonary Disease; ACS: Acute Coronary Syndromes; APACHE II: Acute Physiology and Chronic Health Evaluation; SOFA: Sequential Organ Failure Assessment; pH: potential of hydrogen; PaCO_2_: arterial carbon dioxide partial pressures; PaO_2_: arterial oxygen partial pressures; FiO_2_: fraction of inspired oxygen; PaO_2_/FiO_2_: oxygenation index; WBC: white blood cell; CRP: C-reactive protein; PCT: procalcitonin; ALT: alanine aminotransferase; AST: aspartate aminotransferase; BUN: blood urea nitrogen; PEAR1: platelet endothelial aggregation receptor 1; *P* values were calculated by chi-square test, Fisher exact test, Student’s *t*-test, or Mann–Whitney *U* test, **P* <0.05 indicates statistical significance.

**Table S2.** **The primary antibody of Western blot**

| **Antibody** | **Source** | **Cat No** |
| --- | --- | --- |
| Anti-CD31 | Abcam | ab182981 |
| Anti-CD31 | Abcam | ab7388 |
| Anti-HIF-1α | Abcam | ab1 |
| Anti-HIF-1α | Abcam | ab179483 |
| Anti-HIF-1α | CST | 48085S |
| Anti-Flag Tag | Proteintech | 66008-4-Ig |
| Anti-His Tag | Proteintech | 66005-1-Ig |
| Anti-GST Tag | Proteintech | 10000-0-AP |
| Anti-HA Tag | Proteintech | 66006-2-Ig |
| Anti-IgG | Proteintech | 30000-0-AP |
| Anti-HK2 | Proteintech | 66974-1-Ig |
| Anti-PFKP | Proteintech | 13389-1-AP |
| Anti-PKM2 | Proteintech | 15822-1-AP |
| Anti-LDHA | Cell Singaling | 2012S |
| Anti-AARS1 | Proteintech | 17394-1-AP |
| Anti-AARS1 | Proteintech | 67909-1-Ig |
| Anti-PEAR1 | RD | AF7607 |
| Anti-PEAR1 | Abmart | PHH9765 |
| Anti-Acetyllysine | PTM Bio | PTM-105RM |
| Anti-L-Lactyl Lysine | PTM Bio | PTM-1401RM |
| Anti-L-Lactyl-Histone H3 (Lys18) | PTM Bio | PTM-1406RM |
| Anti-L-Lactyl-Histone H3 (Lys18) | PTM Bio | PTM-1427RM |
| Anti-Acetyl-Histone H3 (Lys18) | PTM Bio | PTM-114RM |
| Anti-TBP | Proteintech | 66166-1-Ig |
| Anti-Phospho-STAT3 | Cell Singaling | 9145S |
| Anti-c-MYC | Proteintech | 10828-1-AP |
| Anti-Phospho-NF-κB p65 | Cell Singaling | 3031S |
| Anti-ZO-1 | Abcam | ab276131 |
| Anti-VE-cadherin | Proteintech | 27956-1-AP |
| Anti-Occludin | Proteintech | 27260-1-AP |
| Anti-Claudin1 | Proteintech | 28674-1-AP |
| Anti-Importin subunit alpha | Abcam | ab307438 |
| Anti-Histone-H3 | Proteintech | 17168-1-AP |
| Anti-beta-actin | Proteintech | 81115-1-RR |
| Anti-GAPDH | Proteintech | 60004-1-Ig |
| CoraLite488-conjugated Goat Anti-Mouse IgG | Proteintech | SA00013-1 |
| CoraLite488-conjugated Goat Anti-Rabbit IgG(H+L) | Proteintech | SA00013-2 |
| CoraLite594-conjugated Goat Anti-Rabbit IgG(H+L) | Proteintech | SA00013-4 |
| CoraLite594-conjugated Goat Anti-Mouse IgG(H+L) | Proteintech | SA00013-3 |
| Mouse Anti-Rabbit lgG HRP (avoid heavy chain) | Abmart | M21006 |
| HRP-conjugated Affinipure Goat Anti-Rabbit IgG(H+L) | Proteintech | SA00001-2 |
| HRP-conjugated Affinipure Goat Anti-Rat IgG(H+L) | Proteintech | SA00001-15 |
| HRP-conjugated Affinipure Rabbit Anti-Sheep IgG(H+L) | Proteintech | SA00001-16 |
| HRP-conjugated Affinipure Goat Anti-Mouse IgG(H+L) | Proteintech | SA00001-1 |

**Table S3. Primers used for quantitative real-time RT-PCR**

| **Gene** | **Primer sequence** |
| --- | --- |
| *Pear1* (mouse） | Forward: ACCTCTCCCGTGACCCATAA  Reverse: TGCCAAGTGCTCATGTTCCT |
| *Hif1a* (mouse） | Forward: TGGAGATGCTGGCTCCCTAT  Reverse: TGTGCTCATACTTGGAGGGC |
| *Myc* (mouse） | Forward: GATCAGCAACAACCGCAAGT  Reverse: CGTTCCTCCTCTGACGTTCC |
| *Stat3*（mouse） | Forward: ACCACGAAAGTCAGGTTGCT  Reverse: AGGCTGCCGTTGTTAGACTC |
| *Rela* (mouse） | Forward: AGACACAGATGATCGCCACC  Reverse: GGCTTGGGGACAGAAGTTGA |
| *Hk2* (mouse） | Forward: ACGAGCTCTCTCTCAACCCT  Reverse: ATTCCCCTTGTCTTGAGGCG |
| *HK2* (human） | Forward: GATTGCCTCGCATCTGCTTG  Reverse: GCTCCAAGCCCTTTCTCCAT |
| *Pfkp* (mouse） | Forward: GCAACTGCAATGTAGCCGTC  Reverse: GCGAATCCTTCAAAGCCGTC |
| *PFKP* (human） | Forward: ATCATCGGTGGATTCGAGGC  Reverse: TTGGACACAGTAGCGGGAAC |
| *Pkm* (mouse） | Forward: TTCTCTACCGTCCTGTTGCG  Reverse: GTAAGCGTTGTCCAGGGTGA |
| *PKM* (human） | Forward: AGAAAGGTGCCGACTTCCTG  Reverse: GCTCGACCCCAAACTTCAGA |
| *Ldha* (mouse） | Forward: GTCCAGCGAAACGTGAACAT  Reverse: CCAAGCCACGTAGGTCAAGA |
| *LDHA* (human） | Forward: ACCGTGTTATTGGAAGCGGT  Reverse: CTCCATGTTCCCCAAGGACC |
| *Tjp1* (mouse） | Forward: TTGAGCAGCCGTCATACAGG  Reverse: CGAGGTTGGTAGGGCTGTTT |
| *Cdh5* (mouse） | Forward: AAGACATCCGAGTGGGCAAG  Reverse: CTGTACTCGCCCTGCATGAT |
| *Ocln* (mouse） | Forward: CCACCCCCATCTGACTATGC  Reverse: TCTGGGTATGATCGCTTGCC |
| *Cldn1* (mouse） | Forward: TATGACCCCTTGACCCCCAT  Reverse: TTGTTTTCCGGGGACAGGAG |

**Table S4. The potential upstream transcriptional regulators of glycolytic genes**

| **HK2** | **PFKP** | **PKM2** | **LDHA** |
| --- | --- | --- | --- |
| HIF-1α | HIF-1α | HIF-1α | HIF-1α |
| c-Myc | c-Myc | c-Myc | c-Myc |
| NF-κB | NF-κB | SP1 | SP1 |
| FOXO1 | FOXO3 | NF-κB | AP-1 |
| CREB1 | E2F1 | STAT3 | NF-κB |
| STAT3 | STAT3 | USF2 | STAT3 |
| E2F1 | USF1 | YY1 | USF2 |
| USF1 | Creb1 | ATF2 | CREB1 |
| AP-2 | AP-2 | FOXO3 | TEAD1 |
| ATF3 | YY1 | E2F1 | FOXO3 |

**Table S5 The *Pear1*-promoter sequence**

Mouse: CCGGGAGCCGGGGCTGTCAGGTTGGGCGGCTCTCCCCGTCCACTTCGCAGACAGGCGTGCGGAGGGCTGACCCCACCCGGCCGGGCTCCAGCCTCCCCGCGCGGACTTCCTGCCTGGGCGCGGGCGGAGGCCCACCCCCTTCCTGCCCGC

Human:

CGCCTGGATCTCCCCTCCCCCGGCTCCTGTTTCCTTGTCAAAACTTCCTGCCTTGGCGAGGGCCCGAGTTCCCACCCCCTTCCTGCCCCCCGCCCCTCGGCGCCCCTCCCGGCCCTGCGATCAGCAGCGTCCCGCCTCCCCGCCGCTCCC

 **Figure S1 PEAR1 is Upregulated in Lung Tissue and PMVECs after S-ALI**

A. HPA database shows the expression level of PEAR1 in human tissues.

B-C. H&E staining of the lung tissue sections (B) and lung injury scores (C) at various time points after CLP surgery (n=5 per group). Scale bar: 500 μm (main) and 50 μm (inset).

D-E. ELISA measurement of L-lactate levels in serum and lung tissue of mice at various time points after CLP surgery (n=5 per group).

F. ELISA measurement of inflammatory cytokines (IL-1β, IL-6, and TNF-α) in BALF of mice at various time points after CLP surgery (n=5 per group).

G. WB analysis of cell junction proteins (ZO-1, VE-cadherin, Occludin, and Claudin-1) in lung tissue of mice at various time points of CLP surgery (n=5 per group).

H-I. Changes in cell viability and relative TEER values at various time points and concentrations after LPS stimulated in MPMVECs (n=5 per group).

J-K. WB analysis of cell junction proteins (ZO-1, VE-cadherin, Occludin, and Claudin-1) in MPMVECs after LPS stimulation at different time points (J) and concentrations (K) (n = 5 per group).

L-M. ELISA measurement of L-lactate and inflammatory cytokines (IL-1β, IL-6, and TNF-α) in the supernatant of MPMVECs with or without LPS stimulated (n=5 per group).

N-Q. Correlation analysis of PEAR1 levels with SOFA score, APACHE II score, CRP and PCT in all sepsis patients.

All data were represented as the means ± SD, **P* < 0.05, ***P* < 0.01, ****P* < 0.001, and *****P* < 0.0001; ns, not significant.

 **Figure S2 PEAR1 Promotes Pulmonary Vascular Hyperpermeability and Exacerbates ALI in Septic Mice**

A. Diagram of the constructed model of *Pear1* knockout (*Pear1*^-/-^) mice.

B. Mouse genotypes were identified by PCR using the two pair of primers. The PCR product length was 738 bp for wild-type (WT) mice and 636 bp for *Pear1*^-/-^ mice.

C. Expression of PEAR1 protein in lung tissue lysate of *Pear1*-WT or *Pear1*^-/-^ mice (n=5 per group).

D. Representative IF staining images showing the endothelial tropism of AAV9-sh-*Pear1*-GFP in mouse lung tissues. GFP signals were detected mainly in CD31-positive pulmonary endothelial cells. Nuclei were stained with DAPI. Scale bar, 20 μm.

E. Representative IF staining images of PEAR1 (green) in lung tissues after endothelial-targeted delivery of AAV9-sh-NC or AAV9-sh-*Pear1*. Vascular endothelium was labeled with CD31 (red), and nuclei were stained with DAPI (blue). Scale bar, 20 μm.

F. H&E staining of lung sections and lung injury scores from SHAM+AAV9-sh-NC, SHAM+AAV9-sh-*Pear1*, CLP+AAV9-sh-NC, and CLP+AAV9-sh-*Pear1* mice (n=5 per group). Scale bar, 50 μm.

G-H. Representative images showing EBD extravasation assay in mouse lung tissues from different groups (G). Leakage degrees were quantified by detecting the EBD contents in lung homogenate (H) (n=5 per group).

I. Representative TEM images showing cell junctions on the surface of pulmonary vessels in different groups. Scale bar: 1 μm (main) and 500 nm (inset).

J. Changes in cell viability of MPMVECs after transfection with si-NC or si-*Pear1*, compared to untreated control cells (n = 5 per group).

K. Relative TEER values of MPMVECs transfected with si-NC or si-*Pear1* and subsequent stimulation with or without LPS (n=5 per group).

L. WB analysis of cell junction proteins (ZO-1, VE-cadherin, Occludin, and Claudin-1) in MPMVECs in different groups (n=5 per group).

M. RT-qPCR analysis of cell junction protein mRNA levels in MPMVECs in different groups (n=5 per group).

N. Representative IF staining images of MPMVECs. VE-cadherin (red), Claudin-1 (green), (N) ZO-1 (red), and Occludin (green). Nuclei were stained with DAPI (blue). Scale bar, 20 μm. Quantification of fluorescence intensity was analysed by Image J (n=5 per group).

All data were represented as the means ± SD, **P* < 0.05, ***P* < 0.01, ****P* < 0.001, and *****P* < 0.0001; ns, not significant.

**Figure S3 PEAR1 Regulates Glycolysis in PMVECs in a Model of S-ALI**

A. Volcano plot of DEGs from RNA-seq of MPMVECs transfected with si-NC or si-*Pear1* and subsequent stimulation with LPS (10 μg/mL, 12 h) (n = 3 per group).

B. Volcano plot of differential metabolite based on targeted energy metabolism analysis from MPMVECs in different groups (n=3 per group).

C. Heatmaps of differential metabolite based on targeted energy metabolism analysis from MPMVECs in different groups (n=3 per group).

D. WB analysis of key glycolytic enzymes (HK2, PFKP, PKM2, and LDHA) in HPMECs transfected with si-NC or si-*Pear1* and subsequent stimulation with or without LPS (n=5 per group).

E. RT-qPCR analysis of key glycolytic enzyme mRNA levels in HPMECs in different groups (n=5 per group).

F. ELISA measurement of L-lactate levels in the supernatant of MPMVECs and HPMECs transfected with si-NC or si-*Pear1*/si-*PEAR1* and subsequently stimulated with LPS (n=5 per group).

G-H. WB analysis of key glycolytic enzyme protein levels in lung tissue of WT and *Pear1*^-/-^ mice after SHAM or CLP surgery (n=5 per group).

I-K. Representative IF staining images in mouse lung tissue in different groups. (I) LDHA (red) and PKM2 (green); (J) PFKP (red) and HK2 (green). Nuclei were stained with DAPI (blue). Scale bar, 50 μm. Quantification of fluorescence intensity was analysed by Image J (n=5 per group).

L. ELISA measurement of L-lactate levels in lung tissue of WT and *Pear1*^-/-^ mice after CLP surgery (n=5 per group).

All data were represented as the means ± SD, **P* < 0.05, ***P* < 0.01, ****P* < 0.001, and *****P* < 0.0001; ns, not significant.

**Figure S4 PEAR1 Increases HIF-1α Binding to Importin α via K172 Lactylation to Promote HIF-1α Nuclear Localization**

A. HIF-1α, c-Myc, STAT3, and NF-κB were identified as common potential upstream transcription factors of the glycolytic key enzymes HK2, PFKP, PKM2, and LDHA through intersection analysis.

B. Representative IF staining images of p-STAT3 (green), p-NF-kB p65 (green) and c-Myc (green) in MPMVECs transfected with si-NC or si-*Pear1* and subsequent stimulation with or without LPS (10 μg/mL for 12 h). Nuclei were stained with DAPI (blue). Scale bar, 20 μm.

C. Representative IF staining images of HIF-1α (green) in HPMECs in different groups. Nuclei were stained with DAPI (blue). Scale bar: 20 μm (main) and 4 μm (inset).

D. AlphaFold structural prediction showed that PEAR1 comprises an extracellular domain (19-754 aa) and an intracellular domain (776-1034 aa).

E. Co-IP was performed to examine lactylation of HIF-1α in LPS-stimulated HPMECs transfected with si-NC or si-*PEAR1* (10 μg/mL for 12 h).

F. Co-IP was performed to examine lactylation of HIF-1α in LPS-stimulated HPMECs transfected with His-*HIF1A*-WT or His-*HIF1A*-K172R (10 μg/mL for 12 h).

G. The sequences around HIF-1α K172 from different mammal species were conserved. HIF-1α K172 conservative lysine residues are marked in red.

H. ECAR was measured after overexpressed His-*Hif1a*-WT or His-*Hif1a*-K172R in LPS-stimulated MPMVECs with or without *Pear1* silencing. And basal glycolysis, glycolytic capacity, and glycolytic reserve were presented as bar graphs in different groups (n=3 per group).

I. WB analysis of cytoplasmic and nuclear HIF-1α expression in LPS-stimulated HPMECs in different groups.

J. WB analysis of HK2, PFKP, PKM2, and LDHA in LPS-stimulated HPMECs in different groups (n=5 per group).

K. ELISA measurement of L-lactate levels in the supernatant of MPMVECs and HPMECs transfected with His-*Hif1a*-WT/His-*HIF1A*-WT or His-*Hif1a*-K172R/His-*HIF1A*-K172R and subsequently stimulated with LPS (n=5 per group).

All data were represented as the means ± SD, **P* < 0.05, ***P* < 0.01, ****P* < 0.001, and *****P* < 0.0001; ns, not significant.

 **Figure S5 PEAR1 Mediates the Binding of AARS1 to HIF-1α, thereby Regulating HIF-1α Lactylation**

A. Co-IP was performed to examine the lactylation of HIF-1α in MPMVECs transfected with control vector or HA-tagged *Aars1* overexpression plasmid, subsequent stimulation with or without LPS (10 μg/mL for 12 h).

B-C. Co-IP was performed to examine the interaction between AARS1 and HIF-1α in MPMVEC transfected with control vector or Flag-tagged *Pear1* overexpression plasmid, subsequent stimulation with or without LPS (10 μg/mL for 12 h).

D. Co-IP was performed to examine the interaction between AARS1 and HIF-1α in

HPMECs transfected with si-NC or si-*PEAR1* and subsequent stimulation with LPS (10 μg/mL for 12 h).

E-F. Molecular docking models predicting the interaction interfaces between the intracellular domain of PEAR1 and AARS1 (E) or HIF-1α (F). The enlarged dashed boxes show the predicted hydrogen-bonding residues within the PEAR1 intracellular region.

G. Co-IP was performed to examine the interaction between AARS1 and exogenously expressed proteins from the overexpressed truncated plasmid *Pear1* (Δ1011-1023 aa) in MPMVECs stimulated with LPS (10 μg/mL for 12 h).

H. Co-IP was performed to examine the interaction between HIF-1α and exogenously expressed proteins from the overexpressed truncated plasmid *Pear1* (Δ1011-1023 aa) in MPMVECs stimulated with LPS (10 μg/mL for 12 h).

I. Co-IP was performed to examine the interaction between AARS1 and exogenously expressed proteins from the overexpressed plasmid (Flag-*Pear1*-WT) or single-site mutant plasmids (G1014A, H1015A, P1019A, and R1022A) in MPMVECs stimulated with LPS (10 μg/mL for 12 h).

J. Co-IP was performed to examine the interaction between HIF-1α and exogenously expressed proteins from the overexpressed plasmid (Flag-*Pear1*-WT) or single-site mutant plasmids (H1011A, Y1016A, V1021A, R1022A, and H1023A) in MPMVECs stimulated with LPS (10 μg/mL for 12 h).

**Figure S6 Glycolysis-Derived Lactate Promotes PEAR1 Expression through H3K18la during S-ALI**

A. WB analysis of H3K9la, H3K14la, H3K27la, H4K8la, and H4K12la in lung tissues from SHAM and CLP groups (n=5 per group).

B-C. WB analysis of H3K18la in HPMECs with or without LPS stimulation (10 μg/mL for 12 h) (n=5 per group).

D. Distribution profiles of H3K18la and H3K18ac at annotated genomic regions in MPMVECs with or without LPS stimulation (10 μg/mL for 12 h) from CUT&Tag analysis (n=3 per group).

E. Heat maps of the genome occupancy of H3K18la ± 3 kb flanking transcription start sites in HPMECs with or without LPS stimulation (10 μg/mL for 12 h) from CUT&Tag analysis (n=3 per group).

F. IGV tracks for *PEAR1* from CUT&Tag analysis in HPMECs.

G. ChIP-qPCR assays of H3K18la occupancy rates in the promoter region of *PEAR1* in HPMECs with or without LPS stimulation (10 μg/mL for 12 h) (n=5 per group).

All data were represented as the means ± SD, **P* < 0.05, ***P* < 0.01, ****P* < 0.001, and *****P* < 0.0001; ns, not significant.

 **Figure S7 E-selectin Binding Peptide-Modified Liposomes Carrying *Pear1*-Targeted Small Interfering RNA Ameliorate ALI in Mice with Polymicrobial Sepsis**

A. The polydispersity indices (PDIs) of Lips@si-*Pear1* and EBP-Lips@si-*Pear1* (n=3 per group).

B. The zeta potential of Lips@si-*Pear1* and EBP-Lips@si-*Pear1* (n=3 per group).

C. Cell viability was assessed by CCK-8 assay in MPMVECs at various time points and concentrations after application with EBP-Lip@si-*Pear1* (n=5 per group).

D. Representative IF staining images of PEAR1 (green) and EBP-Lips@si-*Pear1* (red) in MPMVECs at various time points after application with EBP-Lip@si-*Pear1* (150 μg/mL) (n=5 per group). Nuclei were stained with DAPI (blue). Scale bar, 20μm.

E. H&E staining of various organs sections after the application of EBP-Lips@si-*Pear1* (n=5 per group). Scale bar Scale bar: 500 μm (main) and 50 μm (inset).

F-H. Representative flow cytometry plots showing EBP-Lips@si-*Pear1* signals in CD31-positive endothelial cells (F), CD326-positive epithelial cells (G), and F4/80-positive macrophages (H). the percentages of double-positive cells are indicated in each quadrant. CD31, endothelial cell marker; CD326, epithelial cell marker; F4/80, macrophage marker.

I-K. Representative flow cytometry plots showing Lips@si-*Pear1* signals in CD31-positive endothelial cells (I), CD326-positive epithelial cells (J), and F4/80-positive macrophages (K);

L. WB analysis of key glycolytic enzymes (HK2, PFKP, PKM2, and LDHA) in septic mouse lung tissues after treatment with or without EBP-Lip@si-*Pear1* (n=5 per group).

M. ELISA measurement of L-lactate levels in septic mouse lung tissues after treatment with or without EBP-Lip@si-*Pear1* (n=5 per group).

N. WB analysis of H3K18la expression levels in septic mouse lung tissues after treatment with or without EBP-Lip@si-*Pear1* (n=5 per group).

All data were represented as the means ± SD, **P* < 0.05, ***P* < 0.01, ****P* < 0.001, and *****P* < 0.0001; ns, not significant.

**Clinical ethics**

**Animal Ethics**
